# Spatial Correlations Drive Long-Range Transport and Trapping of Excitons in Single H-Aggregates: Experiment and Theory

**DOI:** 10.1021/acs.jpclett.3c03586

**Published:** 2024-03-01

**Authors:** Alberto Carta, Bernd Wittmann, Klaus Kreger, Hans-Werner Schmidt, Thomas L. C. Jansen, Richard Hildner

**Affiliations:** † Materials Theory, Department of Materials, ETH Zürich, 8093 Zürich, Switzerland; ‡ Spectroscopy of Soft Matter, University of Bayreuth, 95440 Bayreuth, Germany; ¶ Macromolecular Chemistry and Bavarian Polymer Institute, University of Bayreuth, 95440 Bayreuth, Germany; § Zernike Institute for Advanced Materials, University of Groningen, 9747 AG Groningen, The Netherlands

## Abstract

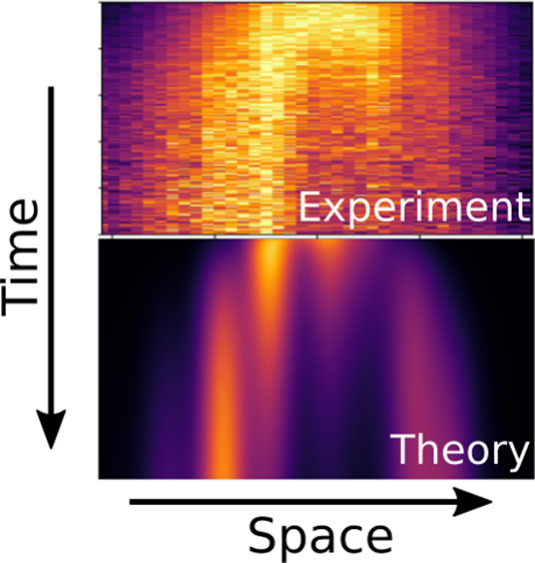

Describing long-range energy transport is a crucial step, both toward deepening our knowledge on natural light-harvesting systems and toward developing novel photoactive materials. Here, we combine experiment and theory to resolve and reproduce energy transport on pico- to nanosecond time scales in single H-type supramolecular nanofibers based on carbonyl-bridged triarylamines (CBT). Each nanofiber shows energy transport dynamics over long distances up to ∼1 μm, despite exciton trapping at specific positions along the nanofibers. Using a minimal Frenkel exciton model including disorder, we demonstrate that spatial correlations in the normally distributed site energies are crucial to reproduce the experimental data. In particular, we can observe the long-range and subdiffusive nature of the exciton dynamics as well as the trapping behavior of excitons in specific locations of the nanofiber. This trapping behavior introduces a net directionality or asymmetry in the exciton dynamics as observed experimentally.

The transport of excitation energy (excitons) in assemblies of organic chromophores is key in understanding biological phenomena such as the light-harvesting mechanisms in photosynthesis,^1^ in understanding processes in photoactive materials,^2^ as well as in the development of potential applications, e.g., for white-light emission..^3^ In particular, the ability to predict and tailor the transport properties of molecular systems that sustain exciton transport is crucial in the design and optimization of novel devices. In the last years, substantial effort has been dedicated to resolve and study energy transport properties of supramolecular nanostructures,^4−6^ in which organic chromophores are tightly packed with a well-defined arrangement. Due to the relevance in biological systems and the potential for technological advancement, a key goal is to ultimately identify systems with long-range (>100 nm) energy transport and to accurately model and understand the underlying mechanisms on a molecular scale.

In general, the dynamics of energy transport in supramolecular nanostructures is dictated by the interplay of electronic interactions between the chromophores and electronic/structural disorder:^1,5−10^ On the one hand, intermolecular electronic coupling favors delocalization of excitons over the aggregate and promote transport. On the other hand, electronic/structural disorder localizes excitons and slows down transport. For strong disorder excitons can become trapped and localized in “*outlier*” states with very low energy in the excited-state energy landscape of the nanostructures.^11^ Often, the description of such trapping exploits heavy-tailed Lévy-type disorder of the site energies, which severely limits achievable transport distances.^11−13^

A difficulty in theoretical modeling of energy transport in real systems is that transport typically takes place in the intermediate regime, in which the magnitude of the electronic coupling is comparable to the strength of electronic disorder. Due to this relatively large disorder in this regime, most models are not able to reproduce long-range exciton transport^8,10,14−17^ observed experimentally.^18−24^ Recently, it has been suggested that spatial correlations of site energies can, to some extent, mitigate the effect of disorder on (short-range) energy transport in amorphous layers of organic molecules.^25^ Such correlations can result from a combination of steric effects and π-stacking interactions of molecules that favor configurations in which molecules are locally aligned.^25^ However, the effect of spatial correlations on long-range energy transport in well-defined supramolecular assemblies has not been investigated yet, although their large impact on photophysics and optical spectra has been known for decades.^26,27^

In a combined experimental and theoretical approach, we show here that spatial correlations in electronic disorder enable long-range energy transport in supramolecular nanofibers. Experimentally, we resolve the energy transport in single nanofibers based on carbonyl-bridged triarylamines (CBT) on pico- to nanosecond time scales and observe transport distances up to ∼1 μm despite pronounced exciton trapping. We are able to reproduce those results with a simple Frenkel exciton Hamiltonian using spatially correlated Gaussian-distributed electronic disorder and describing the exciton dynamics by the Pauli master equation including interactions with a surrounding bath. In addition, we theoretically model our results including coupling to intramolecular vibrations and for different descriptions of the intermolecular electronic coupling. We find that our model is not sensitive to those latter modifications.

We prepared supramolecular nanofibers based on two different compounds, s-CBT^18^ and CBT-NIBT;^19^ see Figure S1 in the Supporting Information. Both compounds have an aromatic core based on a carbonyl-bridged triarylamine (CBT) and bulky, flexible peripheries appended to the CBT-core via three amide linkages as common motifs. Directed hydrogen bonding between the amide groups drives the self-assembly of these compounds into nanofibers with cofacially stacked CBT cores. Along this H-type stack of cores we demonstrated long-range energy transport at room temperature for both compounds.^18,19,28^ The preparation of spatially isolated, single nanofibers with lengths of several μm was reported previously^18,19^ (see the Supporting Information, section *Experimental Methods and Materials*, for details on preparation and Figure S2 for widefield PL images).

To resolve the spatiotemporal exciton dynamics in spatially isolated, single nanofibers based on these CBT derivatives at room temperature, we employ time-resolved detection-beam scanning using a home-built optical microscope (see section *Experimental Methods and Materials*, Supporting Information). With this approach we create an initial exciton population on a nanofiber by a tightly focused excitation pulse (spot size ∼350 nm full width at half-maximum, fwhm). Exciton transport gives rise to a spread of this population with time along the long axis of a nanofiber, along which the CBT-cores are stacked cofacially (Figure 1(a)). We detect this spread of the exciton population via the broadening of the photoluminescence (PL) signal on time scales of pico- to nanoseconds via time-correlated single-photon counting (for excitation conditions, spatial resolution of the excitation, and detection path, see the Supporting Information, section *Experimental Methods and Materials*). To visualize the time dependence of energy transport along the nanofibers’ long axis, we create spatiotemporal PL intensity distributions from those data by normalizing the PL at each point in time.^18^

**Figure 1:**
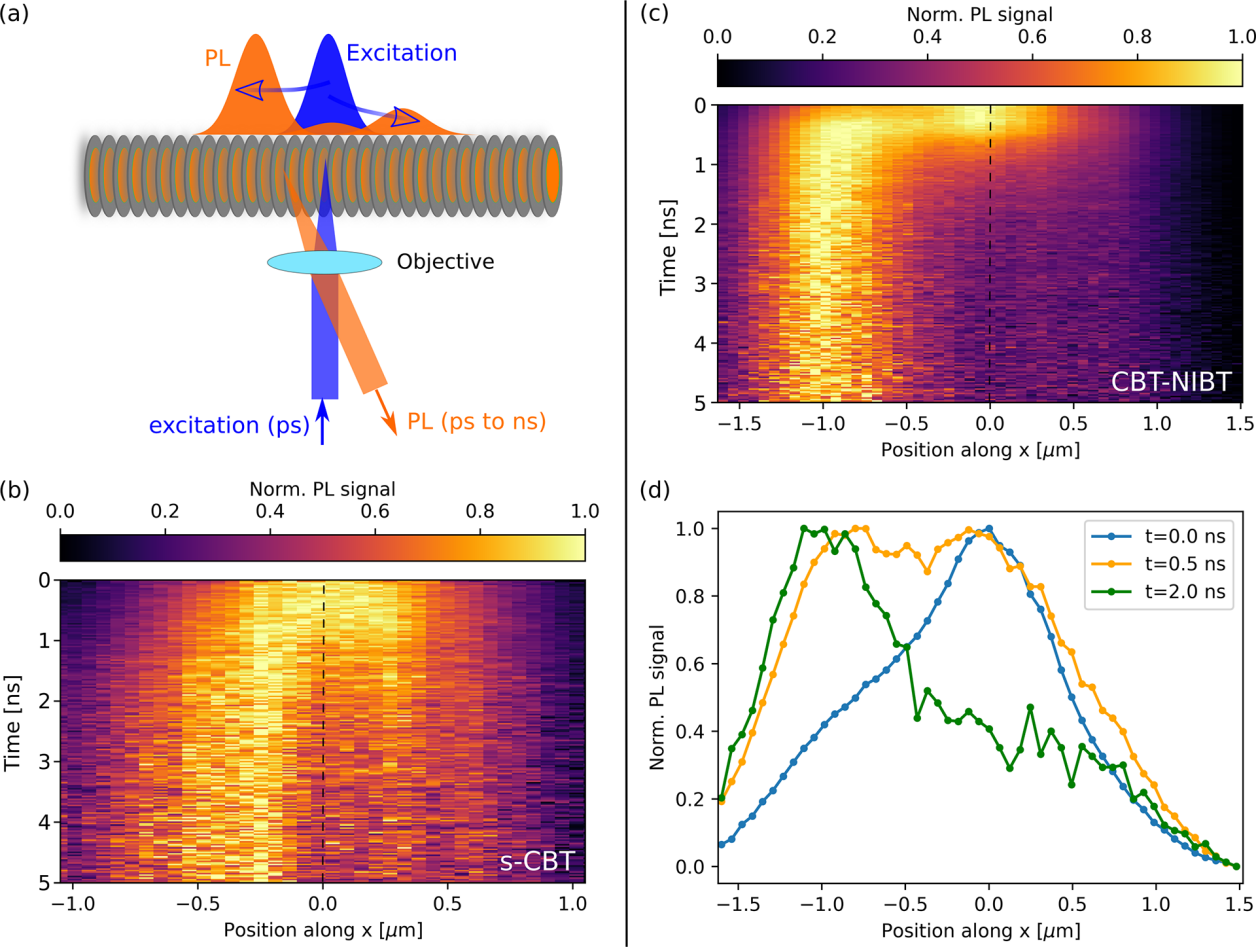
Long-range exciton transport and exciton trapping observed in single supramolecular nanofibers based on CBT derivatives. (a) Concept of the experiment: An initial exciton population (blue, top) is created by a tightly focused excitation beam on a H-type nanofiber with cofacially stacked CBT cores. This population spreads in space along the nanofiber as a function of time (orange) and is detected via its PL signal on pico- to nanosecond time scales. (b,c) Examples of asymmetric PL intensity distributions acquired from a single nanofiber based on s-CBT (b) and CBT-NIBT (c), respectively. (d) Normalized PL profiles extracted from the data on CBT-NIBT in (c) at 0 ns (blue), 0.5 ns (orange), and 2 ns (green) after creating the initial exciton population.

Examples of spatiotemporal PL intensity distributions from two different single supramolecular nanofibers based on s-CBT and CBT-NIBT are shown in Figure 1(b,c), respectively. The position *x* = 0 μm indicates the center of the initial exciton population (the center of the excitation spot) that is created at time *t* = 0 ns. Within some 100 ps after excitation we observe PL from positions several 100 nm, up to ∼1 μm, away from the initial excitation spot, which highlights the long-range nature of transport in our nanofibers. Note that in previous work we have reported on symmetric transport along both directions of single nanofibers.^18^ Remarkably, however, for ca. 10% of nanofibers (6–7 out of ∼60) of each compound (highly), asymmetric transport is found as the data in Figure 1(b,c) show; i.e., transport along one direction is favored. For example, in Figure 1(b) there is a clear trend toward the left of the nanofiber. This observation suggests the presence of some asymmetry in the local excited-state energy landscape around the excitation spot that drives transport predominantly into this specific direction. Yet, transport in the opposite direction (to the right) is not fully suppressed. Importantly, the “delayed” population of exciton states to the left and right of the initial excitation spot is visible in the non-normalized time-correlated single-photon counting traces at *x* ≠ 0 μm as an initial rising component (see the section *Additional Data Sets* of the Supporting Information).

The second example of an asymmetric PL intensity distribution in Figure 1(c) is striking in two aspects: First, exciton transport occurs largely toward one direction and results in a PL signal about 1 μm to the left of the excitation spot. Second, this transport occurs quickly within some 100 ps after excitation, and then excitons appear trapped at a fixed position on the nanofiber (Figure 1(d)). This behavior seems to point to the presence of a specific site or small number of closely spaced sites on this nanofiber with (very) low-lying energy, at which excitons are quickly trapped without being able to escape within their lifetime. We note that although trapping appears to be more pronounced for the specific CBT-NIBT-based nanofiber in Figure 1(c) as compared to the s-CBT-based nanofiber in Figure 1(b), the general features observed for asymmetric transport are largely independent of the compound (for further examples of PL intensity distributions, see the section *Additional Data Sets*, Supporting Information).

The data on single nanofibers in Figure 1 suggest that each nanofiber possesses a unique excited-state energy landscape and that energy transport is strongly influenced by this landscape in terms of direction as well as time scale of transport. Since many excitation–transport–emission cycles are required to build up the PL intensity distributions, excitons can still probe different transport pathways within single nanofibers, leading to a signal at *x* > 0 μm even for the case of strong trapping at *x* ∼ −1 μm observed in Figure 1(c). Notably, despite such pronounced trapping, transport still occurs over substantial distances along a nanofiber, in agreement with the high exciton diffusivities of up to ∼1 cm^2^/s (at *t* = 1 ns) that we have reported recently for this system.^18^

To gain deeper insights into long-range exciton dynamics and the mechanism behind trapping in our H-type nanofibers, we model the system with the Frenkel(−Holstein) exciton formalism,^7,29^ including static electronic disorder and coupling to a bath. For a nanofiber comprising *N* electronically interacting chromophores, here corresponding to the cofacially stacked CBT cores, the Frenkel exciton Hamiltonian reads
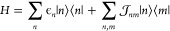
1Here the ket |*n*⟩ represents a single exciton localized on the *n*-th chromophore with local site energy ϵ_*n*_.  is a Frenkel-type coupling that accounts for the intermolecular electronic coupling between sites *n* and *m*. If we restrict our consideration to the nearest-neighbor coupling (*m* = *n* ± 1), the sign of  determines the concavity of the energy dispersion: H-type aggregates have , while for J-type aggregates .^30,31^ We note that in our code we implemented the more general Frenkel–Holstein model Hamiltonian,^7^ which also includes the effect of vibrations. In the case of the one-particle approximation, which is the basis set we adopt to make the problem computationally tractable, mapping back to the Frenkel model (setting *n*_vib_ = 0) leads to rescaling of the effective intersite coupling  by the square of the Franck–Condon factor *f*_00_ that is taken from experimental data.^18^ In terms of the true intersite coupling *J*_*nm*_ we have  (where |*f*_00_|^2^ ∼ 0.5697; see the Supporting Information, section *Frenkel–Holstein Hamiltonian* and section *Basis sets*). It is worth mentioning that we did not find fundamentally different results in the transport properties when including vibrations. Therefore, all results presented in the main text use the simpler Frenkel exciton model described by eq 1, which can more easily be applied to large systems due to the smaller Hilbert space required.

To introduce static electronic disorder in the system we follow the work of Knapp.^26^ We draw the site energies ϵ_*n*_ from a multidimensional normal distribution:
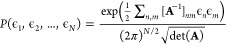
2where the matrix elements of the covariance matrix **A** are written as
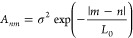
3Here σ is the standard deviation of the individual site energies and *L*_0_ is the characteristic correlation length (in units of chromophores) which determines the decay of the covariance of the Gaussian disorder over several chromophores. For *L*_0_ = 0, all sites are spatially uncorrelated and their energy is drawn independently from the same Gaussian distribution with standard deviation σ. For the limit *L*_0_ → *∞* (correlation length much larger than the nanofiber), all site energies within one nanofiber are identical and the disorder is purely interfiber. In the intermediate case, *L*_0_ is greater than 0 but much smaller than the total nanofiber length. In this situation, the excited-state energies are locally smooth on length scales of *L*_0_, but they can still vary strongly along a single nanofiber.

We now turn to describing the exciton dynamics in our system, including interaction with a surrounding bath, which represents all types of interactions between the nanofiber and the environment. We adapt a formalism described in previous work^13,32−35^ to our case. More formally, we look at the evolution of the reduced density matrix ρ_*νμ*_ of our aggregate, which is obtained from the total density matrix after tracing out the bath.^35^

Under the Born–Markov approximation we make use of the exciton eigenstates of the Frenkel Hamiltonian eq 1 including static disorder as the basis and we consider the scattering between the eigenstates induced by the bath.^33,35^ In this context, the diagonal elements of the exciton density matrix ρ_*μμ*_ represent the probability that the eigenstate μ is populated, while the off-diagonal terms ρ_*μν*_ (with μ ≠ ν) take the meaning of the coherences between the exciton states. Since we are interested in exciton dynamics on pico- to nanosecond time scales, which is much greater than typical coherence times in supramolecular nanostructures (10–100 fs),^36−38^ we further simplify the problem by employing the secular approximation.^35^ This allows us to neglect any coherence effect and concentrate our efforts on the evolution of the diagonal term in the density matrix.

Under these simplifications, the time evolution of each eigenstate population *P*_μ_ ∝ ρ_*μμ*_ can be written in terms of the Pauli master equation:^33,35^
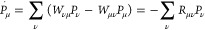
4where  denotes the rate of change of the population and *W*_*νμ*_ represent the scattering rates between eigenstates,^32,35^ which we have rewritten in terms of the update matrix *R*_*μν*_. For the scattering rates we take the expression derived from Fermi’s Golden Rule for scattering with a single environmental phonon,^33^ yielding
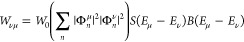
5Here, the prefactor *W*_0_ is a positive real number which controls the strength of the exciton scattering. Φ_*n*_^μ^ is the *n*-th component of the μ-th eigenvector of the aggregate Hamiltonian; so the second term  represents the overlap integral of the exciton probabilities. This term suppresses scattering between states that are further apart along a nanofiber and favors hopping between neighboring states. The third term *S*(*E*_μ_ – *E*_ν_) is the spectral density of the bath and depends on the details of the environment and its coupling to the system. The final term in eq 5, *B*(*E*_μ_ – *E*_ν_), represents the thermal occupation (thermal weight) of the environmental phonons, which is responsible for driving the exciton dynamics toward thermal equilibrium.

The formal solution to the Pauli master equation, eq 4, reads
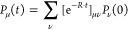
6Knowing the update matrix *R*_*μν*_ of the system, we can compute the time evolution for any given initial state *P*_ν_(0) by numerical matrix exponentiation. For all details on the regime of validity of this model and for the subtleties related to the numerical implementation (such as the specific form of the spectral density and thermal weight), we refer to the Supporting Information (section *Computational details*).

We now have all tools at our disposal to start simulating the exciton dynamics in single nanofibers. In the simulations presented here, we set up a linear chain of 6000 sites (chromophores), which corresponds to a length of ∼2 μm for a nanofiber, given the π-stacking distance of CBT chromophores of about 0.33 nm along the nanofibers’ core.^18^ We employ the Frenkel Hamiltonian with a nearest-neighbor electronic coupling of *J* = 0.1 eV and an average transition energy for the nanofibers of Ω_00_^*^ = 2.3344 eV, which resemble experimentally determined values.^18^ To account for static disorder and spatial correlations, the site energies are drawn according to the distribution in eq 2 and the correlation length defined by eq 3. All numerical parameters for the simulation of the propagation are summarized in Table 1. In Table S1 of the Supporting Information, we report the parameter regime fitted to the spectral data of nonaggregated, molecularly dissolved CBTs and of their nanofibers.

**Table 1:** Summary of Parameters for the Numerical Calculations^a^

Symbol	Description	Value(s)
*J*	Unscaled nearest neighbor intersite coupling	0.1 eV, or ∼ 807 cm^–1^
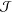	Rescaled nearest-neighbor intersite coupling	0.0569 eV, or ∼ 459 cm^–1^
*k*_*B*_*T*	Thermal energy (room temperature)	0.026 eV, or ∼ 210 cm^–1^
*W*_0_	Scattering prefactor	1 eV, or ∼ 8065 cm^–1^
σ	Static disorder	0.05–0.1125 eV, or ∼ 403–907 cm^–1^
*L*_0_	Correlation length	0–100 sites

a See also section *Fit of the parameters* in the Supporting Information for more details.

We numerically diagonalize the resulting Hamiltonian and simulate a propagation of excitons that are initially (at τ = 0 ns) spatially distributed according to a Gaussian distribution around the central (0th) chromophore of a nanofiber. The standard deviation of this distribution is chosen to be 420 monomers, which translates into a fwhm of 990 monomers or ∼350 nm to emulate the initial population of excitons by confocal excitation in the time-resolved detection-beam scanning measurements (Figure 1). This initial exciton population is time-evolved by the repeated application of eq 6. The resulting profiles at every time step are then convoluted with a Gaussian function with standard deviation of 220 monomers (∼75 nm, corresponding to a fwhm of ∼520 monomers or ∼180 nm). Although this width is smaller than the spatial resolution of our detection system (fwhm ∼ 450 nm; see section *Experimental Methods and Materials* in the Supporting Information), it is still comparable to the experimental situation and it allows us to highlight and visualize the effects of a disordered energy landscape on exciton diffusion and trapping in a clearer way.

The prefactor *W*_0_ enters in our model as a multiplicative factor only in eq 4 and eq 5. While the value of *W*_0_ will have quantitative effects over the time scales at which the dynamics of the system occurs, the results are robust with respect to this choice, both in terms of the asymmetry of the propagations and in terms of subdiffusive behavior. For these reasons, we selected a value of *W*_0_ = 1 eV (∼10*J*) which yields the best agreement with experimental data and falls within the range of values for *W*_0_ found in the literature.^32,34^

To quantify the evolution of the excitons within a nanofiber, specifically for different degrees of disorder, we perform several calculations for different choices of the parameters (σ, *L*_0_). We time-evolve the diagonal occupations of the eigenstates *P*_μ_(*t*) by employing eq 6 and then transform back to a site representation using the relation
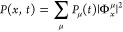
7where *x* is the site number (position of a chromophore) and *P*(*x*, *t*) represents the site occupation at time *t*. This allows us to track the spatial evolution of the exciton population at every time step.

Figure 2 shows the propagations up to *t* = 6 ns for four different single nanofibers with selected sets of parameters (σ, *L*_0_). Panel (a) shows a typical propagation for the case of low static disorder σ = 0.625*J* without intersite correlations *L*_0_ = 0. The exciton population evolves rather uniformly along both directions of the nanofiber, with the profile of the propagation maintaining a fairly symmetric shape. In panel (b) we raise the static disorder σ in the absence of intersite correlations and observe essentially no change in the propagation profiles. The exciton population appears to be stuck in the middle of the nanofiber (*x* = 0 μm), where it was initially placed. Panel (c) shows the effect of an increased correlation length *L*_0_ = 20 with slightly increased disorder σ = 0.75*J* (as compared to (a)). In this case, we find a strong asymmetry in the evolution of the propagation profile. The exciton population mostly drifts toward the right side of the nanofiber and eventually becomes trapped around 0.4 μm. To a lesser extent, excitons also propagate to the left and become ultimately trapped at −0.5 μm. If we raise the correlation length to *L*_0_ = 50 (panel (d)), the asymmetry becomes more pronounced. The excitons drift within less than 500 ps toward a preferential position at 0.33 μm and localize there. To a lesser extent exciton diffusion to the left to −0.4 μm and further to −0.66 μm within ∼1 ns is also observed. For more examples of propagations for each pair of (σ, *L*_0_) we refer to the Supporting Information, Figure S6. Overall, we thus find that “switching on” site energy correlations reproduces our experimental data very well (compare Figure 1(b,c) to Figure 2(c,d)) in terms of all key aspects, i.e., transport distances, asymmetry of the propagations, and trapping, for values of disorder close to experimental numbers.^18^

**Figure 2:**
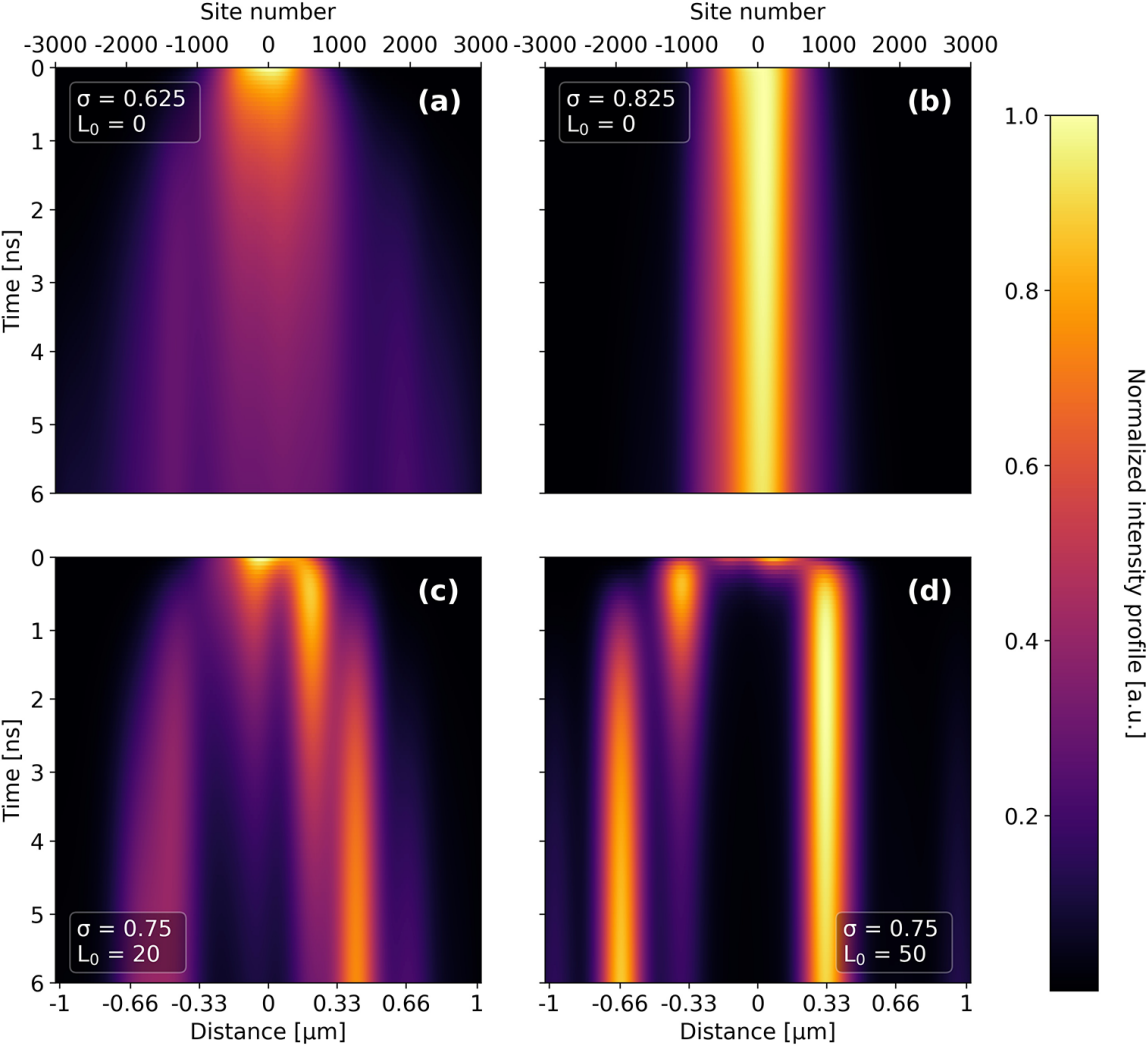
Effect of static disorder σ and correlation length *L*_0_ on the exciton dynamics in single nanofibers. Propagation profiles are shown for (a) moderate-low disorder and no intersite correlations, (b) increased disorder without intersite correlations, (c) correlated moderate disorder, and (d) strongly correlated moderate disorder. The disorder is given in units of the electronic coupling *J*; see Table 1.

To assess quantitatively and more systematically the nature of the exciton dynamics, we evaluate the second moment of the spatial exciton distribution as a function of time, defined as
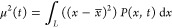
8where *x̅* is it the mean position of the exciton population. The change in the second moment Δμ^2^(*t*) = μ^2^(*t*) – μ^2^(0) provides a measure of exciton diffusion and allows us to determine the nature of the transport. In Figure 3(a) we depict the time evolution of Δμ^2^(*t*) for 100 realizations of disorder (thin lines) for selected pairs of parameters (σ, *L*_0_). The thick lines show the corresponding averages over all 100 realizations. We fitted the Δμ^2^(*t*) curves of each realization *i* for each parameter set between 0 and 1 ns with a monomial of the type Δμ^2^(*t*) = *A*_*i*_*t*^α_*i*_^, with the exciton hopping coefficient *A*_*i*_ and the diffusion exponent α_*i*_. Every simulation yields α_*i*_ < 1 which is indicative of subdiffusive behavior and characteristic of exciton transport in a disordered energy landscape. In the rest of the text, we refer to *A* and α as the average over all realizations of disorder of *A*_*i*_ and α_*i*_, respectively, for each set (σ, *L*_0_). As a general trend, we observe that Δμ^2^(*t*) increases (i.e., both *A* and α increase) with decreasing disorder and fixed correlation length. Increasing the correlation length at fixed static disorder increases the hopping coefficient *A* but lowers α, resulting in an overall increase of Δμ^2^(*t*). This trend is consistent with the notion that decreasing disorder and/or increasing correlation favors exciton delocalization. In turn, a more efficient transport over longer distances is possible.

**Figure 3:**
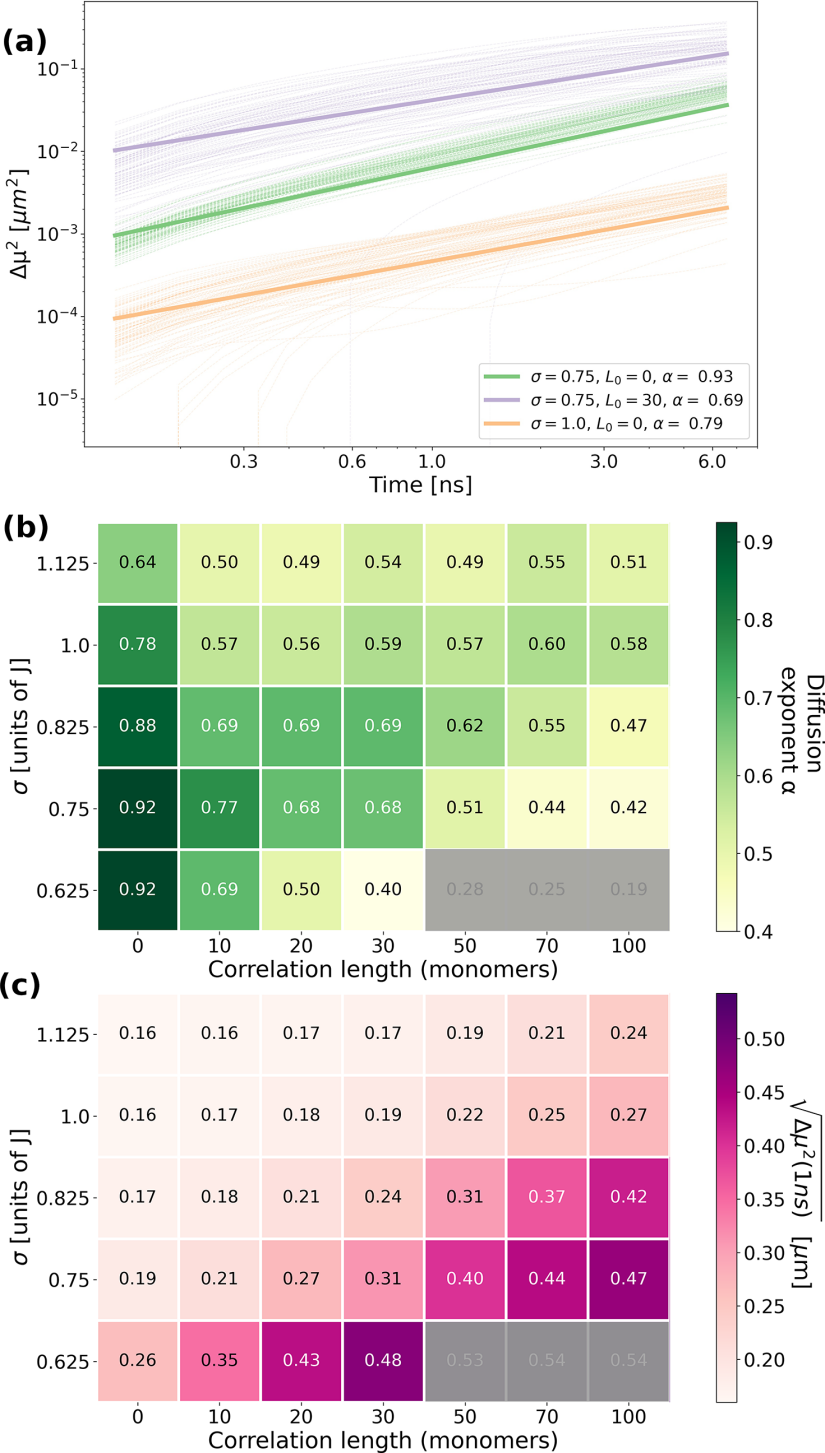
(a) Evolution of the second moment Δμ^2^ of the exciton population distribution for 100 realizations of disorder (thin lines) for different combinations of disorder and correlation length (different colors). The thick lines show the average over all 100 realizations for each combination of σ and *L*_0_. (b) Heatmap of the diffusion exponent α during the first nanosecond. (c) Heatmap of  after 1 ns, which provides a measure for the exciton diffusion length. The areas that are grayed out in (b,c) correspond to very low values of α and should be disregarded, since those arise from boundary effects with the propagation reaching the ends of the nanofiber too rapidly.

These general trends are captured in Figure 3(b,c), which shows a more systematic exploration of the (σ, *L*_0_) parameter space in terms of the diffusion exponent α during the first ns and of  after 1 ns as a measure for the exciton diffusion length. Both quantities are averaged over all realizations of disorder for each set of parameters. The darker colored region in the bottom left of Figure 3(b) corresponds to low σ and low *L*_0_, where the system’s behavior is close to diffusive transport. As either σ or *L*_0_ is increased, the diffusion exponent decreases and the exciton dynamics becomes more and more subdiffusive. The effect of increasing disorder can be understood quite straightforwardly: Due to the nature of static disorder with its Gaussian site energy distribution, some sites close to the initial position will have much lower site energy than an average site. Hence, excitons quickly localize and propagation is confined around the excitation point (see, e.g., Figure 2(b)). The effect of increasing *L*_0_ is less obvious: While a greater correlation length enhances the overall distance traversed by the exciton (as can be seen in higher overall values of  after 1 ns in Figure 3(c)), it also seems to lower the diffusion exponent. The origin of this counterintuitive behavior is that intersite correlations favor the formation of “trapping” domains. As discussed above, the Gaussian distribution of site energies results in some sites with a much lower site energy in the tail of the distribution. If the disorder is spatially correlated, these low-lying sites tend to be surrounded by other sites of similar energy. This creates low-energy regions—“trapping” domains—in the nanofiber where the excitons can delocalize over a number of chromophores of the order of *L*_0_. Yet, these regions tend to be separated by domains with sites of higher energies, thus isolating the “trapping” domains and limiting exciton diffusion (see, e.g., Figure 2(c,d)).

To illustrate this behavior on an example, Figure 4 gives a detailed breakdown of the asymmetric exciton propagation shown in Figure 2(c) for the parameter set σ = 0.75*J* and *L*_0_ = 20, which closest resembles our experimental data in Figure 1 (additional breakdowns for the rest of the panels in Figure 2 are available in the Supporting Information, section *Additional data sets*). In Figure 4(a) we plot the site energies of the chromophores along the nanofiber, while in Figure 4(b) the site energies are depicted as a histogram (blue bars), which nicely follows a Gaussian distribution (black solid line), as well as the density of states (DOS, red bars), which shows a broadening induced by exciton hopping. The effect of spatial correlations becomes evident from the inset in Figure 4(a), which shows an expanded view of the site energies around chomophore 1250 to the right of the center of the nanofiber. Around the (local) minimum at chromophore 1260 the site energies do not vary much, but toward the left and right of this minimum the site energies are substantially higher by more than 0.2 eV. Although excitons will be delocalized to some extent in this minimum with rather smooth site energies, excitons will remain trapped there since the barrier of 0.2 eV vastly exceeds thermal energy. Hence, this local minimum constitutes one of the “trapping” domains of this aggregate (others are highlighted by the colored ellipses in Figure 4(a)). Notably, neither in the DOS nor in the tails of the eigenstate distribution are there features that can be readily identified with “trapping” domains; those are only visible in the site energy distribution in real space; see, e.g., Figure 4(a). The impact of those domains on exciton dynamics is visualized in Figure 4(c) showing three spatial profiles extracted at different times from the data in Figure 2(c): At *t* = 0.07 ns after creating the initial population, the exciton population still has a relatively symmetric distribution with some deviations from the Gaussian shaped excitation toward the right (blue profile). As time proceeds, the excitons spread further (orange profile, *t* = 1.38 ns) and ultimately separate into two distinct regions to the left and right of the initial population (green profile, *t* = 5.99 ns). Notably, the peaks in the time evolution of the propagation profiles correspond to the (very) low-lying domains in the energy landscape of the nanofiber, highlighted in Figure 4(a) by the colored ellipses. These domains are ultimately responsible for slowing down and localizing (trapping) excitons during the propagation. Figure 4(d) shows the evolution of the population in terms of the occupation probabilities of the eigenstates (excitons) of the nanofiber for the same times after excitation as in Figure 4(c). We find that, for the longest time after excitation shown here (*t* = 5.99 ns), the system approaches a Boltzmann distribution (black line) but does not fully reach it. We note that experimentally it is not possible to probe still longer time scales because of the limited exciton lifetime; we therefore did not simulate longer propagations.

**Figure 4:**
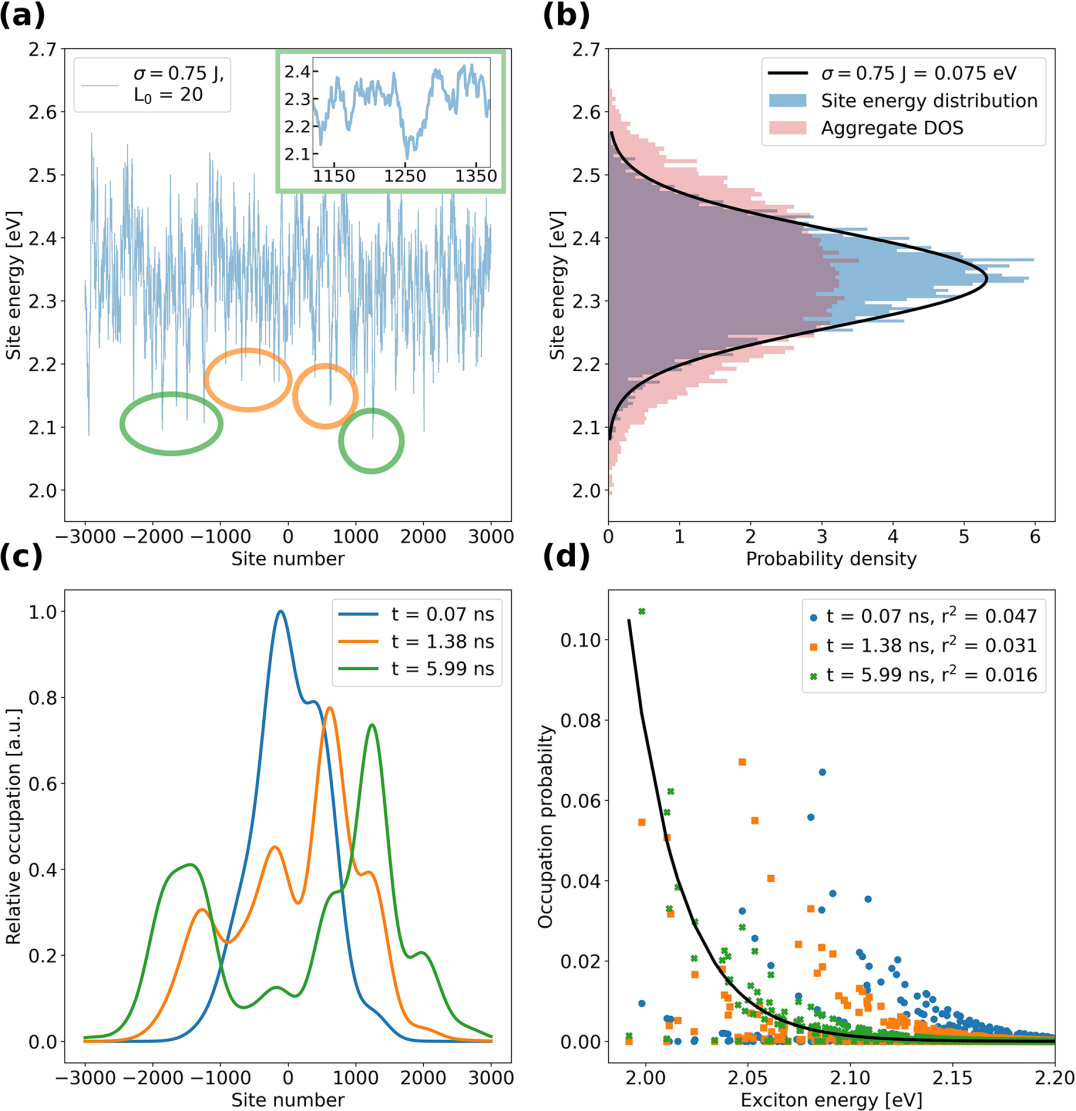
Detailed breakdown of the propagation of the single nanofiber that is presented in Figure 2(c) for the set of parameters σ = 0.75*J* and *L*_0_ = 20. (a) Site energies along the nanofiber. Local low-lying domains are highlighted by colored ellipses. The inset shows an expanded view of the ”trapping” domain around the 1260-th site. (b) Site energy distribution (blue bars), density of state of the aggregate (DOS, red bars), and a Gaussian function with a width of 0.75*J* (black solid line). (c) Spatial profiles of the propagation in Figure 2(c) at different times after excitation, demonstrating the trapping of excitons in the low-energy domains of the nanofiber indicated in panel (a). (d) Same propagations as in panel (c), now in terms of eigenstate populations. The solid black line is a Boltzmann distribution for *k*_*B*_*T* = 0.025 eV, and the legend gives the least-squares error *r*^2^ between the eigenstate population and the Boltzmann distribution, which becomes smaller as time progresses.

These results allow us to gain a better understanding of the two main effects that characterize the dynamics of the system, i.e., the relaxation toward a Boltzmann distributed population and the slowing down of transport due to “trapping” domains. The initial illumination creates higher lying exciton states that then relax from their initial distribution in space and energy toward various low-lying states spread out in the aggregate. This relaxation drives the system toward thermal equilibrium, where the eigenstates of the aggregate are populated according to a Boltzmann distribution. However, the obstacle toward full thermalization is the confinement of the excitons due to the (random) distribution low- and high-energy domains within the energy landscape along the nanofiber: The lower-lying domains trap the excitons and favor their localization to length scales of *L*_0_, while the higher-lying domains act as energy barriers that impede the transport between regions in the aggregate. Finally, we highlight that there is no reason to expect a net directionality of the exciton transport since we use a “symmetric” Gaussian site-energy distribution. In practice, the correlated static disorder breaks the translational symmetry of the nanofiber and creates the low-lying domains, in which excitons become trapped. Note that these domains appear naturally in our model due to the occurrence of low-energy sites in the tail of Gaussian site-energy distributions for a large number of sites per nanofiber. In turn, those domains are responsible for the asymmetric profiles of the evolution of the exciton population observed in the experiments and simulations.

In a joint experimental–theoretical endeavor, we gained important insights into mechanisms of energy transport in supramolecular assemblies of organic molecules. We performed measurements on a well-defined model system, single H-type supramolecular nanofibers, which allowed us to resolve the specific exciton dynamics on pico- to nanosecond time scales along each nanofiber with its unique realization of the excited-state energy landscape. Using a simple Frenkel exciton Hamiltonian, we have successfully reproduced all key aspects of the experimental data, i.e., long transport distances of up to ∼1 μm, the subdiffusive nature of the transport as well as trapping of excitons in a few domains that comprise several neighboring chromophores at low energies in the energy landscape of a nanofiber. This trapping imposes a net directionality (asymmetry) in the energy transport, even though a defined gradient in the energy landscape to steer transport is absent.^39^ Notably, a normal (Gaussian) distribution of site energies, accounting for static electronic disorder, is sufficient to model this trapping of excitons; skewed Lévy distributed energy landscapes^11−13^ are not required. The key factor is that we allow for spatial correlations in the site energies over some tens of chromophores along a nanofiber. Although having been used in theoretical modeling of the photophysics of assemblies of organic molecules,^26,27,40,41^ the role of such correlations to enable long-range exciton transport has not been investigated yet.

We found that the interplay between the static disorder and correlation length lies at the heart of long-range subdiffusive transport and exciton trapping: The systems’ tendency toward thermalization is the driving force that guides the exciton population toward the low-lying states within the Gaussian distribution. At the same time, spatial correlations in the static disorder create an alternation of low-energy and high-energy domains with an extent of the order of the correlation length along the nanofibers, thus creating locally smoother energy landscapes over those length scales. The precise correlation length controls the occurrence and the extension of low-lying domains, which slow down the propagation, and trap and steer excitons toward a preferential direction.

The best match between experiments and simulations, in terms of the (static disorder σ, correlation length *L*_0_) parameter space, is found for the region around σ ∼ 0.75*J* = 0.075 eV and *L*_0_ ∼ 20, compare, e.g., Figures 1 and 2 and Table S1 in the Supporting Information. For a given σ the precise value of *L*_0_ to observe asymmetric transport depends on the local energy landscape, i.e., the specific realization of disorder around the excitation spot. Note that in recent work we determined a value for static disorder of σ ∼ 0.125 eV = 1.25*J* and electronic coupling of *J* = 0.1 eV from absorption and PL spectra of nanofibers in solution.^18^ Based on results from simulations (Figures 3(b,c) and 2) asymmetric transport is not expected for this large disorder. However, it is important to realize that we studied here single nanofibers, each of which may feature a smaller σ as compared to the solution (ensemble-averaged) value. Moreover, since we observed asymmetry in the transport only for ca. 10% of nanofibers, this implies that for most of them disorder can in fact be large and no long-range asymmetric transport can be observed. Only a (small) subset of nanofibers that possesses (by chance) a small disorder then shows this particular transport behavior.

Further efforts in modeling of transport processes are needed to quantitatively assess the role of vibrational states in the exciton propagation. While we made a first attempt at including the effect of coupling to intramolecular vibrations in the Frenkel–Holstein model, in the specific case of the CBT-based nanofibers the vibrational modes are not crucial in describing the dynamics. With vibrational energies of ∼1550 cm^–1^ (or 190 meV) the vibrational states are, for the most part, energetically far away from the vibrational ground state, and hence, the more delocalized higher-lying exciton states are hardly thermally excited and do not give significant contributions to the transport. However, vibrations with lower frequencies may affect the transport properties more significantly, if they have significant exciton–phonon coupling and have frequencies comparable to that of the energy gaps between the exciton states relevant for transport.^42−45^ This would potentially facilitate energy transport due to the multiple hopping pathways resulting from the hybridization of excitons with localized phonons. Such a study would open up avenues for the *bottom-up* design of supramolecular systems capable of still faster long-range energy transport.

Finally, it would be interesting to understand the microscopic origin of the spatial correlations in the static disorder of the nanofibers. Energy transport occurs along the cofacially stacked CBT cores of nanofibers based on s-CBT and CBT-NIBT, respectively. We speculate that the side groups appended to the CBT cores (see Figure S1) may form locally ordered structures to provide a locally shared electronic environment for the CBT cores over lengths of some tens of molecules along a nanofiber.^18,40^ Since such correlations are present in nanofibers based on both CBT derivatives that possess different side groups (but identical CBT cores), a shared environment seems to be present as long as the periphery is bulky and flexible enough to be able to arrange into locally aligned configurations. Alternative mechanisms for (local) site-energy correlations have been proposed to be long-range electrostatic interactions between molecules in a nanostructure and charged/polar groups in the surrounding^41^ or charge-permanent dipole interactions.^46^ The latter interactions could in fact be relevant in our nanofibers, since the C_3_-symmetrically positioned amide groups give rise to unidirectional 3-fold intermolecular hydrogen-bonding, which results in macrodipoles along the nanofibers’ core.^47^ The macrodipoles’ interaction with residual charges in the peripheral groups or on the substrate can then cause long-range interactions and possibly site-energy correlations. Multiscale modeling-type calculations combining density functional theory calculations and molecular dynamics^48^ allow us to investigate the influence of the side groups and the substrate and might provide further insights here. Since the spatial correlations in site energies play a key role for enabling long-range transport, as we showed here, it is clearly a very important aspect to address in future work.

## Supporting Information Available

Details about synthesis and preparation of nanofibers as well as about the experimental setup (figure of chemical structures and absorption and PL spectra); additional experimental data and additional data sets from simulations (figures of PL results and propagation profiles and breakdowns); computational details on the numerical implementation of the method, on the treatment of intramolecular vibrations, and different descriptions of intermolecular electronic coupling; fit of the parameters (figure of absorption spectra, table of spectral parameters) (PDF)
