# Supplementary material for: Spatial Correlations Drive Long-Range Transport and Trapping of Excitons in Single H-Aggregates: Experiment and Theory
Source: J Phys Chem Lett. 2024 Mar 1;15(10):2697–707. doi: 10.1021/acs.jpclett.3c03586 (PMC10946646; doi:10.1021/acs.jpclett.3c03586)
Supplement: Supplementary file 1 — jz3c03586_si_001.pdf [file jz3c03586_si_001.pdf]

Supporting Information: Spatial Correlations Drive Long-Range Transport and Trapping of Excitons in Single H-Aggregates: Experiment and Theory

Alberto Carta,*,† Bernd Wittmann,‡ Klaus Kreger,¶ Hans-Werner Schmidt,¶ Thomas L.C. Jansen,*,§ and Richard Hildner*,§

†*Materials Theory, Department of Materials, ETH Zürich, 8093 Zürich, Switzerland*

‡*Spectroscopy of Soft Matter, University of Bayreuth, 95440 Bayreuth, Germany*

¶*Macromolecular Chemistry and Bavarian Polymer Institute, University of Bayreuth, 95440 Bayreuth, Germany*

§*University of Groningen, Zernike Institute for Advanced Materials, 9747 AG Groningen, The Netherlands.*

E-mail: alberto.carta@mat.ethz.ch; t.l.c.jansen@rug.nl; r.m.hildner@rug.nl

Experimental Methods and Materials

Materials

The synthesis of the molecular building blocks s-CBT and CBT-NIBT was published elsewhere.[1–3] Their chemical structures are shown in Fig. S1. Both compounds are based on a C_3-symmetric carbonyl-bridged triarylamine (CBT) as core, which is linked via three amide groups to flexible and bulky side groups, (S)-chiral aliphatic chains (s-CBT) and bithiophene-naphthalimides (CBT-NIBT), respectively. The preparation of spatially isolated, single supramolecular nanofibres based on these compounds was reported previously.[1,4,5] Briefly, for the preparation of supramolecular nanofibres based on s-CBT we added this compound to *n*-dodecane at a concentration of $\sim 40\ \mu$M. After ultrasonication for 15 minutes the dispersion was refluxed for 30 minutes under stirring and was finally allowed to cool to room temperature. Isolated nanofibers were prepared from this dispersion after it was stored for at least 24 hours, diluted to about 4 μM and spin-coated on microscopy coverslips (borosilicate glass, Carl Roth). Supramolecular nanofibres based on CBT-NIBT were prepared by dissolving this compound in *ortho*-dichlorobenzene (*o*-DCB) at a concentration of 7 μM and heating this solution close to the boiling point of *o*-DCB ($\sim$ 180°C). After cooling to room temperature, the solution was diluted to 0.07 μM and spin-coated on microscopy cover slips (see above). The nanofibers are spatially isolated by several μm on the glass substrates, so that we can identify and address single nanofibers by optical microscopy. All spin-coated samples were dried under vacuum prior to microscopic investigations under ambient conditions.

In our recent work we demonstrated that for both compounds the CBT cores stack cofacially via $\pi - \pi$ interactions in combination with directed hydrogen bonding between the amide groups of neighbouring molecules.[1,4] This stacking results in the H-type nature of the nanofibres' cores as evidenced by their optical spectra, see Fig. S1 below. Note that for CBT-NIBT the absorption is a superposition of the absorption of the CBT core and of the

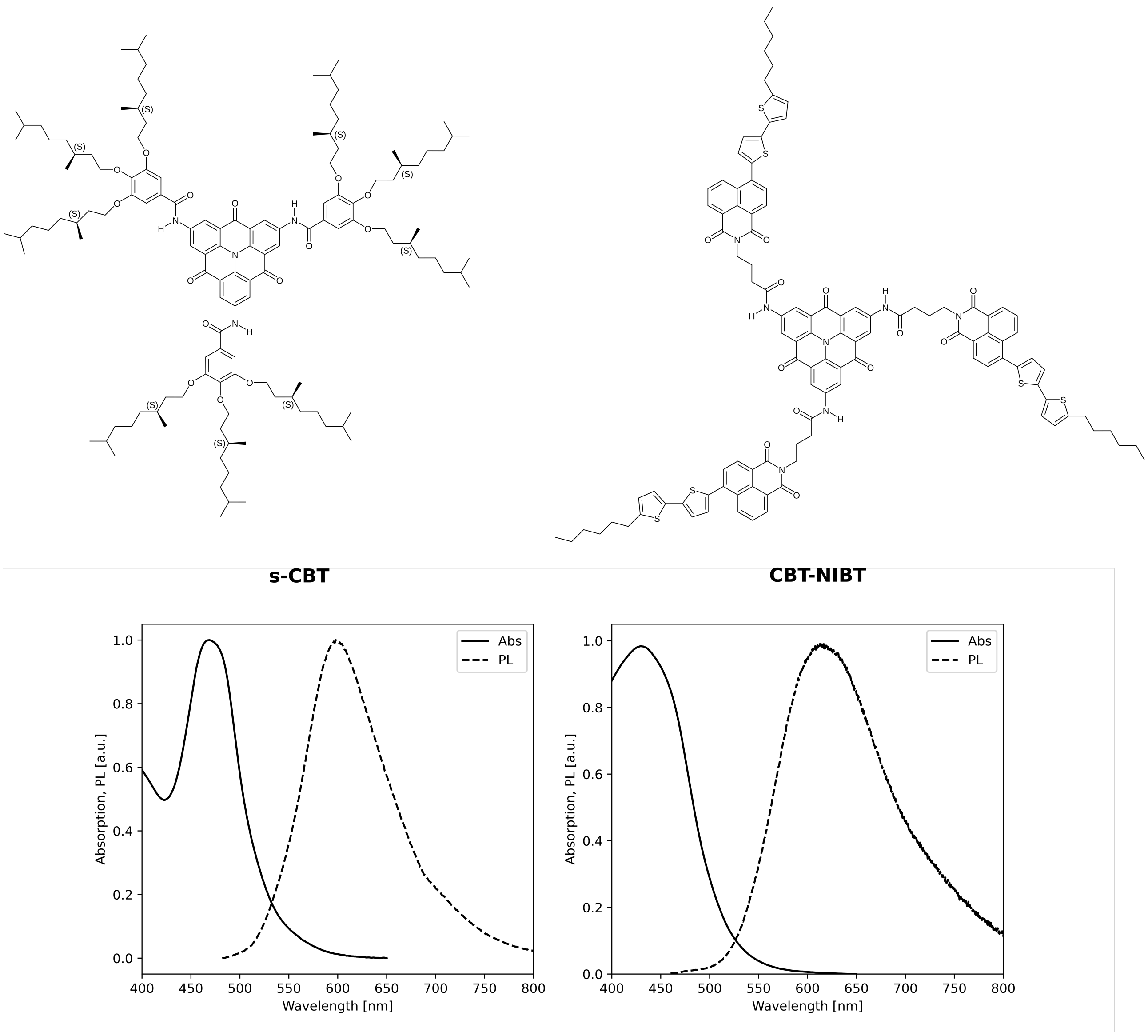

Figure S1: *Top: Chemical structures of the derivative of carbonyl-bridged triarylamine trisamides s-CBT (left) and CBT-NIBT (right). The peripheral groups of s-CBT are three tris((S)-3,7-dimethyloctyloxy) benzoic acid groups and those of CBT-NIBT are three 4-(5-hexyl-2,2'-bithiophene)naphthalimide (NIBT) moieties. Bottom: Absorption (solid) and PL spectra (dashed) of nanofibres based on s-CBT in n-dodecane (left) and on CBT-NIBT in o-DCB (right). Adapted from Refs.* [1,2,4]

peripheral NIBT groups, while the PL exclusively stems from the peripheral NIBT groups populated (after energy transport along the CBT core) via incoherent energy transfer from the CBT core.[1] We also reported excited-state (amplitude-weighted) lifetimes for nanofibres in *n*-dodecane (for s-CBT) of 5.4 ns and in *o*-DCB (for CBT-NIBT) of 2.5 ns.[2,4]

Experimental Setup

To investigate exciton dynamics in isolated supramolecular nanofibres, we employed time-resolved detection-beam scanning using a home-built optical microscope, as described in detail in Refs.[4–6] The microscope can be operated in widefield and in confocal mode: In widefield mode photoluminescence (PL) imaging was performed to identify isolated nanofibres; in confocal mode we acquired spatio-temporal PL data sets of exciton transport in space and time. As excitation source we used a pulsed diode laser operating at 450 nm (LDH-P-C-450B, Picoquant, repetition rate 2.5 MHz, pulse duration 70 ps). After spatial filtering the laser light was directed to the microscope equipped with an infinity-corrected oil-immersion objective with high numerical aperture (NA = 1.45, PlanApo, Olympus). In confocal mode the laser light was tightly focussed to a spot size of $\sim$ 350 nm (FWHM) on the sample, while in widefield mode an additional lens is flipped into the excitation path to illuminate a larger area of around 70 μm in diameter on the sample. A piezo-stage (Tritor 102 SG, piezojena) in closed-loop mode was used to control the sample position with a high precision. The PL from isolated supramolecular nanofibres was collected by the same objective, passed long-pass filters (LP467, AHF) to suppress residual laser light, and was directed to detectors. In widefield mode the PL signal was detected by a scientific CMOS camera (Zyla 4.2, Andor), which allowed to identify isolated nanofibres and to determine their precise orientation on the sample. A single nanofibre was then positioned such that its centre was located in the diffraction-limited spot after switching to confocal mode. To resolve exciton transport and trapping in space and time, we switched (in confocal mode) to a second detection path equipped with a telecentric lens system, a closed-loop piezo scan

mirror (S-335.2.SH, PI) and a single-photon counting photodiode (MPD, Picoquant), which allowed to position the (diffraction-limited) detection spot independently from the excitation spot. The MPD's signal was fed into a time-correlated single-photon counting module (TimeHarp 200, Picoquant) to record PL decays on ps to ns time scales as a function of the detection position for a fixed excitation position. All experiments were performed under ambient conditions at room temperature.

The spatio-temporal data sets acquired from each supramolecular nanofibre were combined into PL intensity distributions, $I(x,t)$. After normalisation at each point in time those distributions visualise how the PL signal evolves in time and space starting from the diffraction-limited excitation spot. In other words, the distributions visualise how an initially local exciton population spreads along the long axis of an individual nanofibre as a function of time after excitation (see Fig. 1b,c in the main text). To estimate the spatial resolution for the detection of the PL signal we have to consider two distinct time regimes in the PL intensity distributions: (i) At $t = 0$ ns we create an initial, roughly Gaussian-shaped exciton population with a FWHM of 350 nm (see above). For the detected PL signal this means that its width is the convolution of two Gaussians with a FWHM of 350 nm, i.e., 495 nm. Moreover, we have to consider that for the excitation we use a wavelength of 450 nm, while the PL is red-shifted. Assuming an 'average' PL wavelength of 575 nm, as estimated from the PL spectra,[1,4] the convoluted width from above must be further multiplied by the ratio of those wavelength. We finally obtain $\sim$ 630 nm. (ii) For times $t > 100$ ps, the PL stems from excitons localised on small domains on the nanofibres comprising only several molecules, which can be assumed to be point-like on length scales of the resolution limit. Hence, the spatial resolution for the PL signal corresponds to a good approximation to the FWHM of the excitation rescaled by the difference in wavelengths (450 nm vs. 575 nm), i.e., $\sim$ 450 nm.

For the acquisition of the spatio-temporal exciton dynamics using the confocal mode the excitation intensities were below $140\,\mathrm{W\,cm^{-2}}$. Based on the absorption at the excitation

wavelength of 450 nm this intensity corresponds to a maximum excitation probability of 0.1 absorbed photons per pulse per 3000 CBT-cores along a column.[4,5] Since we excite singlet excitons at such low density per pulse, singlet-singlet annihilation (of singlets created by the same pulse) does not play a role. Given the repetition rate of 2.5 MHz (400 ns pulse-to-pulse time), singlets created by subsequent pulses also cannot annihilate due to their lifetime of several nanoseconds only.[1,4] Although singlets can convert into triplet excitons via intersystem crossing, and the latter possess a lifetime of $\sim 1\,\mu$s,[5] singlet-triplet annihilation is irrelevant too. Due to the low singlet density, an intersystem crossing yield of less than 85 %,[2] and the long interval between pulses of 400 ns, there is barely triplet population left, when a subsequent pulse arrives. For an in-depth discussion of annihilation processes in our nanofibres and regimes, in which those processes are important, we refer the reader to our recent work.[5]

We also exclude waveguiding and polariton-effects to account for the time-dependent broadening of the PL signal along the nanofibres' long axis. Waveguiding occurs with the speed of light (in medium) and our observed broadening of $\sim 1\,\mu$m would thus occur within a few femtoseconds, which is inconsistent with the broadening within several 100 ps in our data (see Fig. 1(b,c) and Figs. S3 and S4). Polariton formation can be excluded too, since it usually requires a cavity.[7] Although recently 'cavity-free' polariton formation was shown in low-disorder organic nanostructures.[8] Those nanostructures had a diameter of $\sim$ 100 nm, i.e., a diameter of the order of the wavelength of light (within the nanostructures' medium), which can form an effective (low-quality) cavity due to the interface with surrounding media.[9] Our nanofibres, in contrast, possess a diameter of only $\sim$ 4 nm with an ill-defined interface to the surrounding due to the bulky and flexible side groups. Thus light can not be 'confined' within nanofibres to enable strong coupling and polariton formation.

Additional data sets

Experimental data

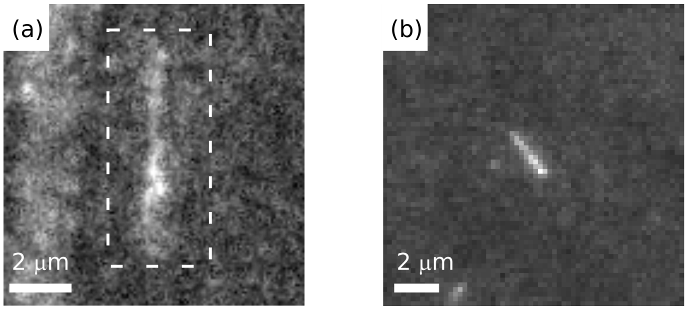

Figure S2: *(a) Widefield PL image of a supramolecular nanofibre based on s-CBT prepared in n-dodecane and deposited on a substrate. The feature in the dashed box represents a single nanofibre; the broader signal at the left are several nanofibres, probably in a side-by-side alignment. (b) Widefield PL image of a supramolecular nanofibre based on CBT-NIBT prepared in o-DCB and deposited on a substrate. Adapted from Refs.* [1,4]

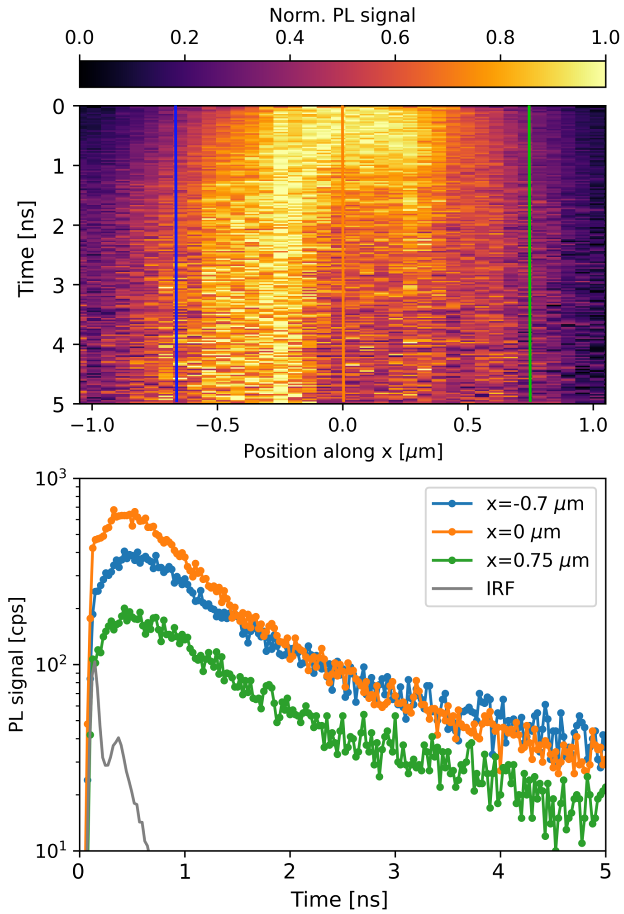

Figure S3: *Top: Spatio-temporal PL intensity distribution from Fig. 1(b) of the main text measured on a nanofibre based on s-CBT, normalised at each point in time. Bottom: PL decay curves extracted at -0.7 μm (blue), 0 μm (orange), and -0.75 μm (green). All PL curves decay on similar (nanosecond) time scales, in accordance with the lifetime measured in solution (see above, Materials section). For the decays at $x \neq 0\,\mu m$ a clear rising component appears, indicating that exciton states away from the centre of the excitation spot are populated with a short $\sim 100\,ps$ delay due to energy transport along the nanofibre's long axis.*

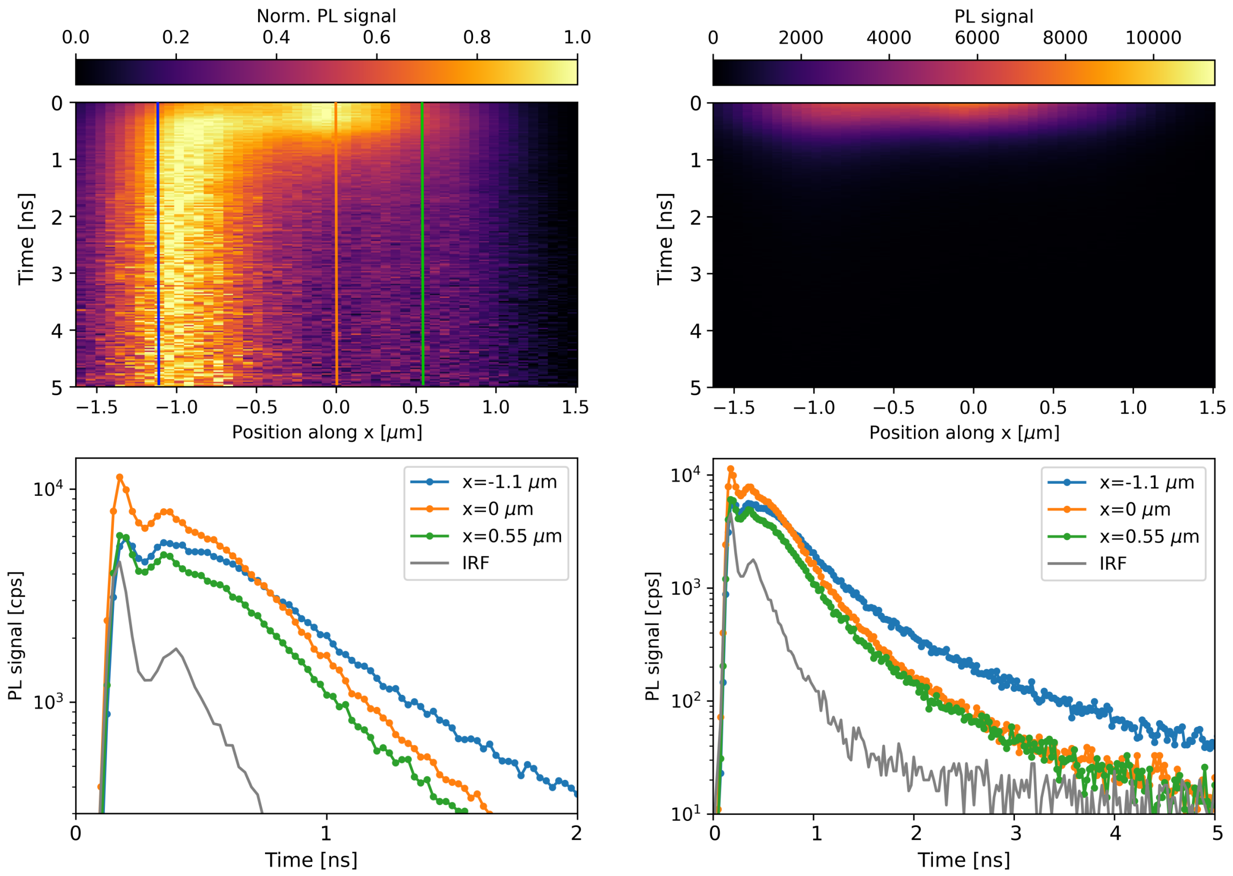

Figure S4: *Top row: Spatio-temporal PL intensity distribution from Fig. 1(c) of the main text measured on a nanofibre based on NIBT-CBT, normalised at each point in time (left). The non-normalised PL intensity distribution (right) shows that the exciton dynamics is not visible without normalisation due to the nanosecond excited-state lifetime, in accordance with the lifetime measured in solution (see above, Materials section). Bottom row: PL decay curves extracted at -1.1 μm (blue), 0 μm (orange), and -1.1 μm (green), showing that the excitons decay always on similar time scales of a few nanoseconds. The left PL decays are zoomed-in on the time and PL signal axis to emphasise the different dynamics on sub-nanosecond time scales for the different positions on the nanofibre, in particular, the rising features appearing at* $x \neq 0\,\mu m$.

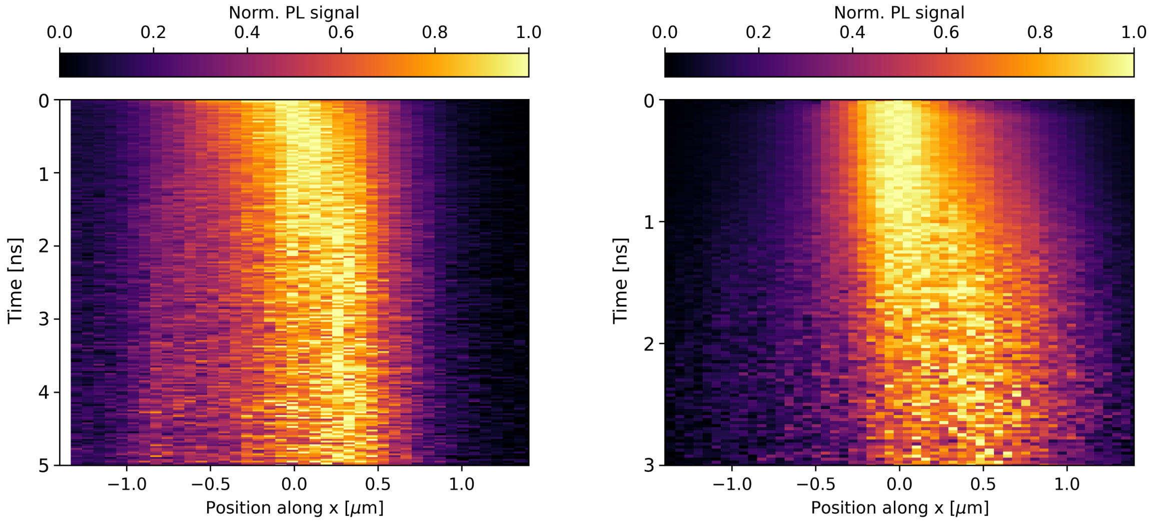

Figure S5: *Additional spatio-temporal PL intensity distributions from nanofibres based on s-CBT (left) and CBT-NIBT (right). Note the different time axis for the CBT-NIBT data set.*

Additional data sets from simulations

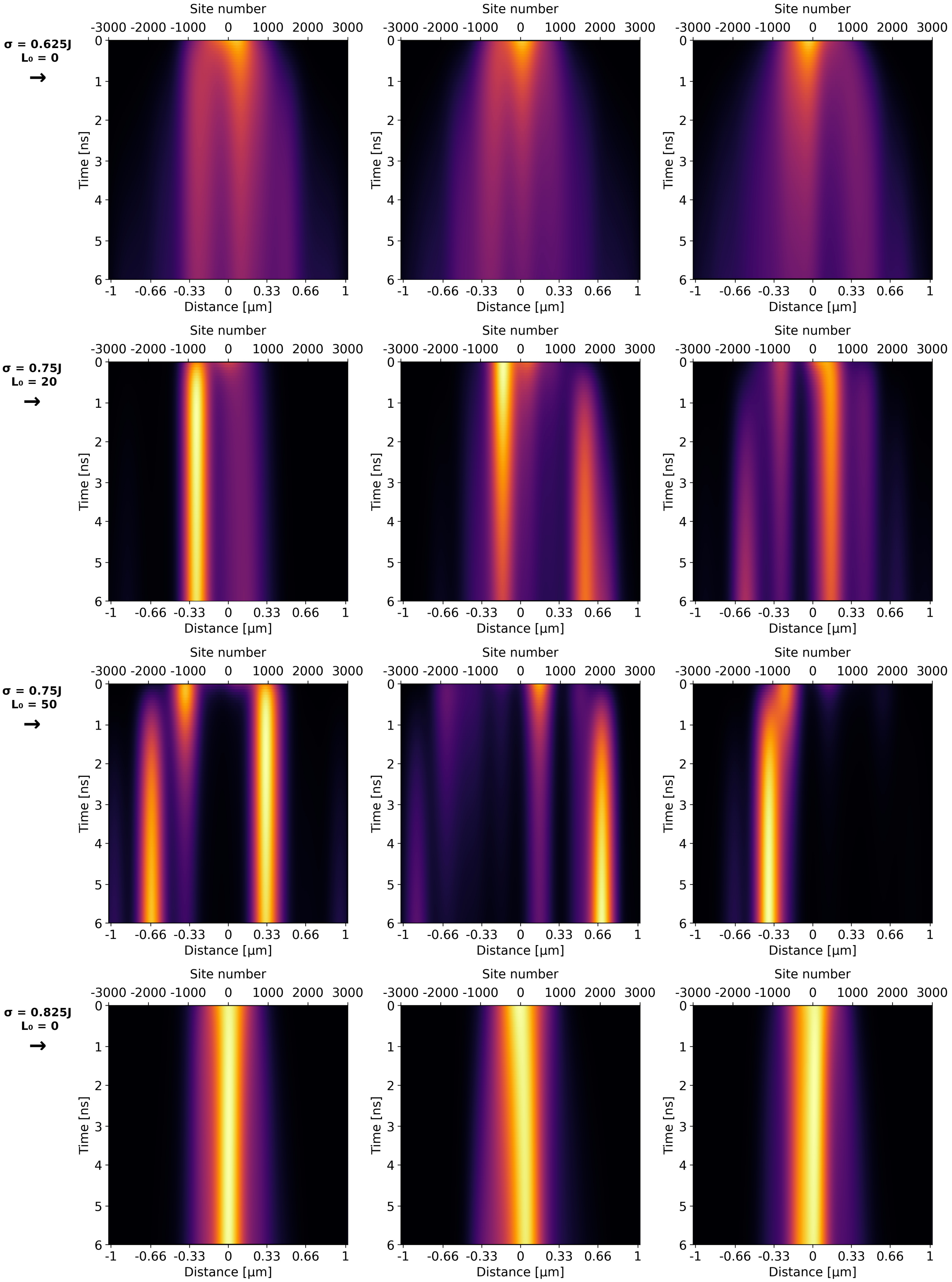

Figure S6: Additional examples of simulations of the propagation profiles for the choices of σ and L_0 given in Fig.2 of the main manuscript. We consider only nearest neighbor hopping $J = 0.1$ eV

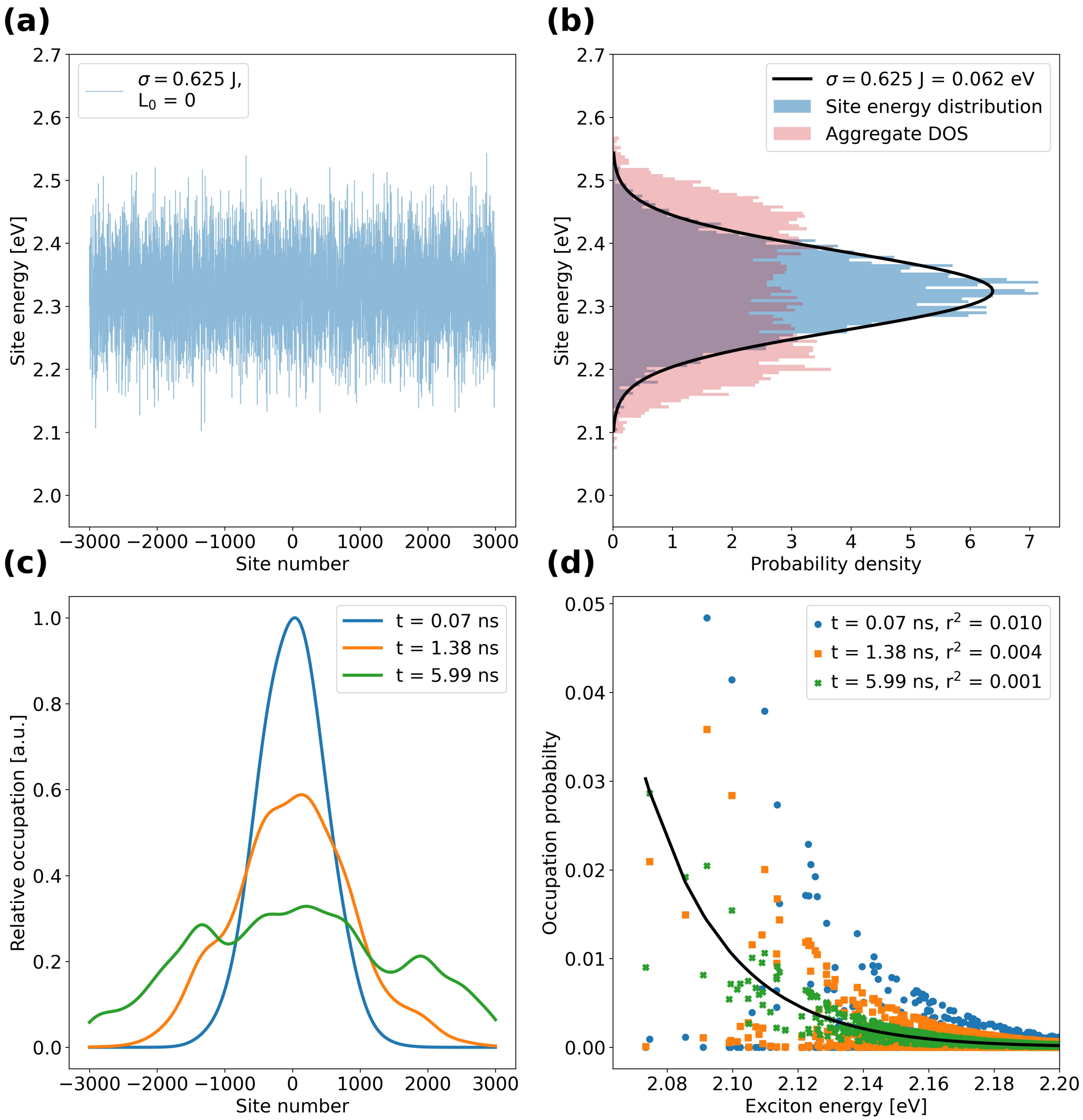

Figure S7: Propagation breakdown for Fig.2(a)

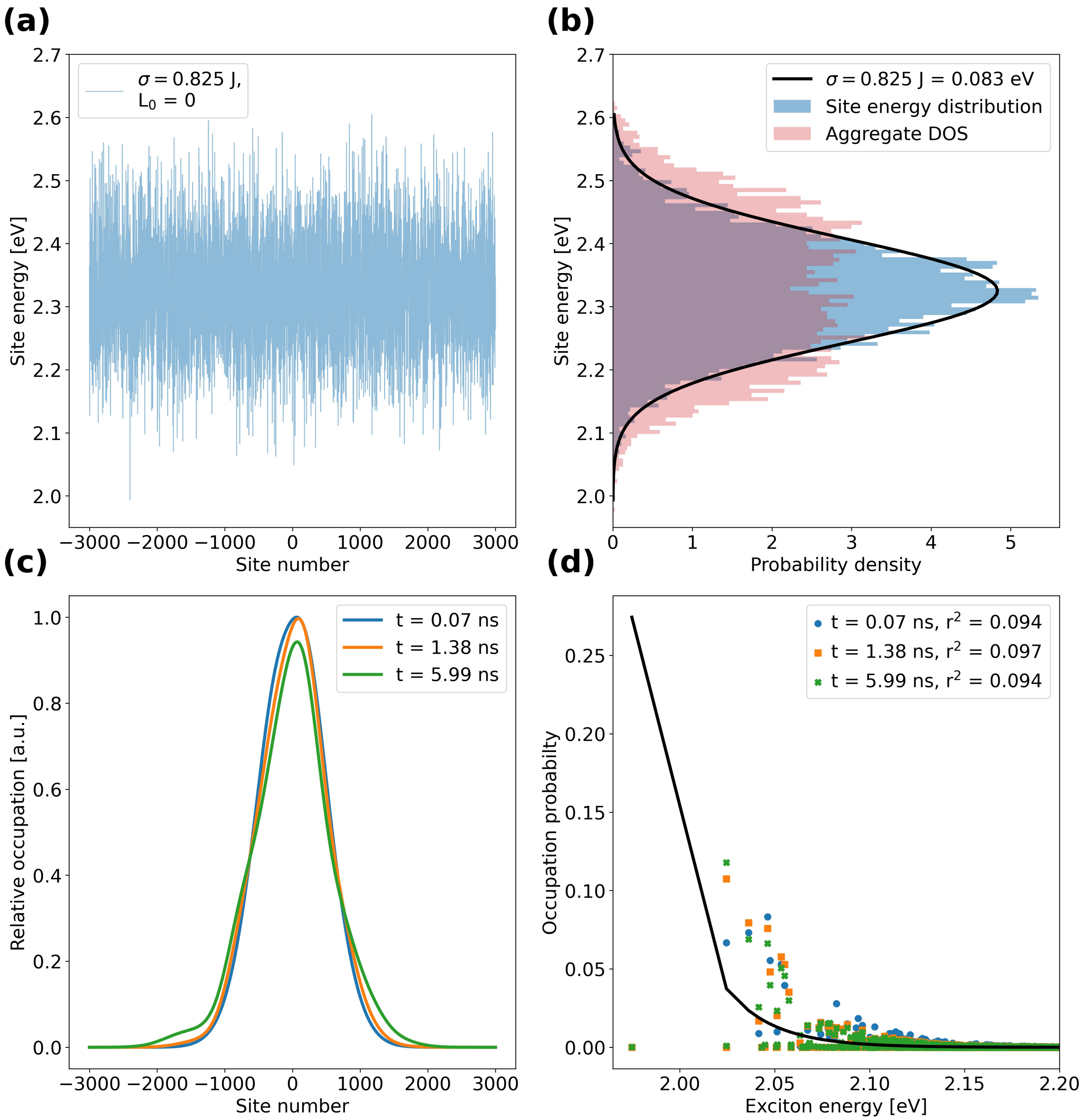

Figure S8: Propagation breakdown for Fig.2(b)

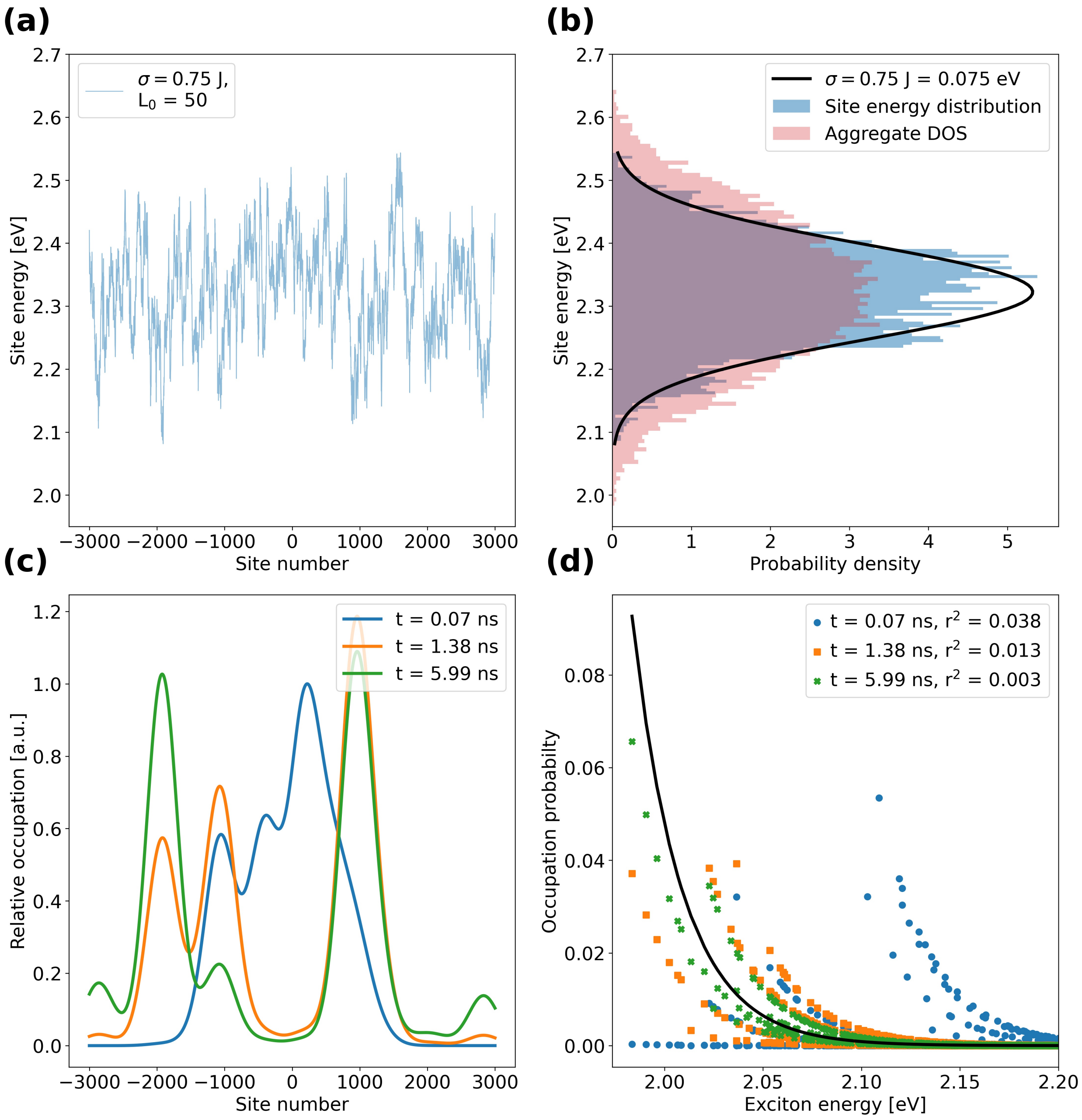

Figure S9: Propagation breakdown for Fig.2(d)

Computational details

Frenkel-Holstein Hamiltonian

The Frenkel exciton Hamiltonian neglects the interplay between the excitons and the localized molecular vibrations (C-C stretching modes, triangle breathing modes, etc.[10–12]). If we want to include these effects, we need to describe the molecular aggregates in the context of the more complicated Frenkel-Holstein model, whose Hamiltonian reads,[10,11,13]

$$\begin{aligned} H = &\sum_n (\epsilon_n + \omega_0 b_n^\dagger b_n) \left|n\right\rangle \left\langle n\right| \\ &+ \sum_{m,n} J_{nm} \left|n\right\rangle \left\langle m\right| \\ &+ \sum_n \lambda\omega_0 (b_n^\dagger + b_n) \left|n\right\rangle \left\langle n\right| + \omega_0 b_n^\dagger b_n, \end{aligned} \tag{S1}$$

Here, the term ϵ_n takes the meaning of the energy of a pure electronic excitation of the n-th chromophore. ω_0 is the energy of an intramolecular vibration, which we assume independent of the position within the aggregate. λ is known as the Huang-Rhys factor and determines the coupling between the vibrational and electronic excitations.[11] b_n and $b_n^\dagger$ are the annihilation and creation operators of a vibrational excitation on the n-th site which obey the commutation rule $[b_m, b_n^\dagger] = \delta_{mn}$. So far, the Hilbert space described is infinite due to the theoretically infinite number of vibrational quanta that could be present on each chromophore, in the next section we describe further simplifications we can employ to make the problem computationally tractable.

Basis sets

The most convenient basis set to tackle the diagonalization of the Hamiltonian in Eq. (S1) is spanned by the exciton-phonon product states:

$$|n,\nu\rangle = \bigotimes_{n} |n\rangle\, |\nu_m\rangle\,, \tag{S2}$$

Where, $|\nu_m\rangle$ indicates the number of vibrations present on the m-th chromophore respectively. Since the basis set of the model described is theoretically infinite, in order to reduce the complexity we restrict the Hilbert space by employing the 'one-particle approximation' (OPA), where both the electronic excitations and the vibrations are assumed to reside on the same chromophore. The basis element in the OPA takes the form:

$$|n,\nu\rangle_{\text{OPA}} = \bigotimes_{n\neq m} |e_n,\nu_n\rangle\, |g_m,0\rangle\,, \tag{S3}$$

Where e_n and g_n represent the excited and ground state of the electronic degree of freedom of a single chromophore. From this point on we set in our notation $|n,\nu\rangle_{\text{OPA}} := |n,\nu\rangle$ unless specified otherwise. In the OPA the Hamiltonian Eq. (S1) reads:

$$H_{OPA} = \sum_{n,m}^{N_{\text{mol}}} \sum_{\nu,\nu'}^{N_{\text{vib}}} (\epsilon_n + \omega_0\nu)\delta_{nm}\delta_{\nu\nu'}\, |n,\nu\rangle\,\langle n,\nu| + J_{nm} f_{0\nu} f_{0\nu'}\, |n,\nu\rangle\,\langle m,\nu'|\,, \tag{S4}$$

Where $f_{0\nu}$ is the Franck-Condon factor ($f_{0\nu}^2 = \exp(-\lambda^2)\lambda^{2\nu}/\nu!$). Given N_{mol} chromophores in an aggregate and truncating the number of possible allowed molecular vibrations to N_{vib}, the complexity of the problem scales as $N_{\text{vib}} \times N_{\text{mol}}$.

For completeness, we also present the basis set chosen for the 'two-particle approximation' (TPA). In this basis set, we allow for an additional vibrational mode to be hosted on one of

the chromophores in the electronic ground state, namely:

$$|n,\nu;m,\nu'\rangle_{\text{TPA}} = |e_n,\nu\rangle \otimes |g_m,\nu'\rangle \otimes \prod_{j\neq n,m} |g_j,0\rangle \,. \tag{S5}$$

Using this basis set, the leading term in the dimension of the Hilbert space scales as $(N_{\text{vib}} \times N_{\text{mol}})^2$. In the following sections, we first employ both the TPA and the OPA to fit the free parameters in Eq. (S1) to the available experimental data. Then, we will restrict ourselves just to the OPA when tackling the exciton transport in order to afford to computationally treat system of several µm in length.

Propagating the dynamics

Expanding on what we wrote in the main text, we write more explicitly here the expression of the scattering matrix R in Eq. (4) of the manuscript:

$$R_{\nu\mu} = \begin{cases} -W_{\nu\mu}, & \text{if } \mu \neq \nu \\ \sum_\eta W_{\eta\mu}, & \text{if } \mu = \nu \,, \end{cases} \tag{S6}$$

where the entries of the W matrix are defined in Eq. (5) of the main text. Concerning the spectral density term, we choose for simplicity an ohmic bath, which is widely used in modeling dissipative quantum systems.[14,15] Its expression reads:

$$S(E) = \frac{|E|}{J}. \tag{S7}$$

We note that in our implementation we have more choice over the specific form of the bath spectral density (super-ohmic form, modulation with exponential cutoff, ...). However, there is only a weak dependency of the overall exciton dynamics on the particular form of the spectral density.[16]

The expression of the thermal weighting of the bath degrees of freedom $B(E)$ reads:[16,17]

$$B(E) = \begin{cases} \left[\exp\left(\frac{E}{k_b T}\right) - 1\right]^{-1} & \text{if } E > 0 \\ 1 + \left[\exp\left(-\frac{E}{k_b T}\right)\right]^{-1} & \text{if } E \leq 0 \end{cases}, \tag{S8}$$

ensuring that the Pauli master equation satisfies the detailed balance condition for the scattering rates $W_{\mu\nu} = \exp\left(-\frac{E_\mu - E_\nu}{k_b T}\right) \cdot W_{\nu\mu}$, and that the population eventually thermalizes to a Boltzmann distribution.[17]

By looking at the formal solution of the Pauli master equation (Eq. (6) of the main text), we can take advantage of the Markovian (memoryless) nature of our system: The time evolution of the exciton populations in energy space depends only on the current state of the system. We then model the dynamics as a continuous Markov process. We define the Markov matrix M through its entries $M_{\mu\nu}$ as:

$$M_{\mu\nu}(\Delta t) = (e^{-R\cdot\Delta t})_{\mu\nu}, \tag{S9}$$

so that the time evolution of the population $P_\mu(t)$ can be expressed as:

$$P_\mu(t + \Delta t) = \sum_\nu M_{\mu\nu}(\Delta t) P_\nu(t). \tag{S10}$$

Given a timestep Δt and an initial state $P_\mu(0)$, this allows to calculate the time evolution of the state by iterative multiplication of the state vector with the Markov matrix.

Numerical implementation

Once all important parameters $(J_{nm}, \lambda, \omega_0, k_B T, W_0, \sigma, L_0, N_{\text{mol}}, N_{\text{vib}})$ are chosen, we write the Hamiltonian in the OPA basis set. If the number of vibrations N_{vib} is set to 1, λ and ω_0 become redundant and the only effect is a rescaling of the inter-site electronic coupling. We then solve the problem using exact diagonalization to extract eigenvalues and eigenvectors of the full Hamiltonian. We proceed to compute the R matrix and perform numerical expo-

nentiation for $\Delta t = 10^5\,\hbar/eV \sim 0.065$ ns to compute the Markov M matrix (we employed the Julia interface to Expokit[18] to numerically evaluate the matrix exponentiation).

As described in the main text, for the initial population $P(0)$ we take a Gaussian profile for the spatial distribution of the excitons centered around the middle of the aggregate with standard deviation of 420 monomers ($\sim$ 150 nm). We then iterate the multiplication of the M matrix and the population vector [Eq. (S10)] to get the population at several time slices and transform the occupations from the basis set of the eigenvectors to the position basis using Eq. (7). The whole process was implemented in Julia and the code is available for download at https://github.com/alberto-carta/CBT_Propagation.

Regime of validity

In this section we briefly address the circumstances under which our model can be considered reliable. We assume the non-radiative decay rates of the excitons Γ to be negligible ($\Gamma \ll W_0$). We also assume no polaronic effects to take place, this is ensured by asking the exciton delocalization length L_D to be much lower than the total length of the aggregate.[19] The delocalization length is defined in terms of the Inverse Participation Ratio IPR_μ of an eigenstate,[19] computed as:

$$\mathrm{IPR}_\mu = \frac{1}{\sum_n |\Phi_n^\mu|^4}, \tag{S11}$$

Here, Φ_n^μ is the eigenvector of the Hamiltonian. L_D is then defined in terms of the mean value of the IPR.[19] In our system, we will be propagating the dynamics of thousands of monomers while a typical value for L_D is between 10 and 70 monomers. We also note that we always consider single particle processes in our treatment, therefore we completely neglect any type of exciton-exciton interaction. Finally, we assume the intensity of emission to be proportional to the local exciton density.

Fit of the parameters

In the following section we discuss the steps we took to fit the numerical values of the parameters needed for the simulation of a realistic nanofiber.

Absorption spectrum of the non-aggregated monomers

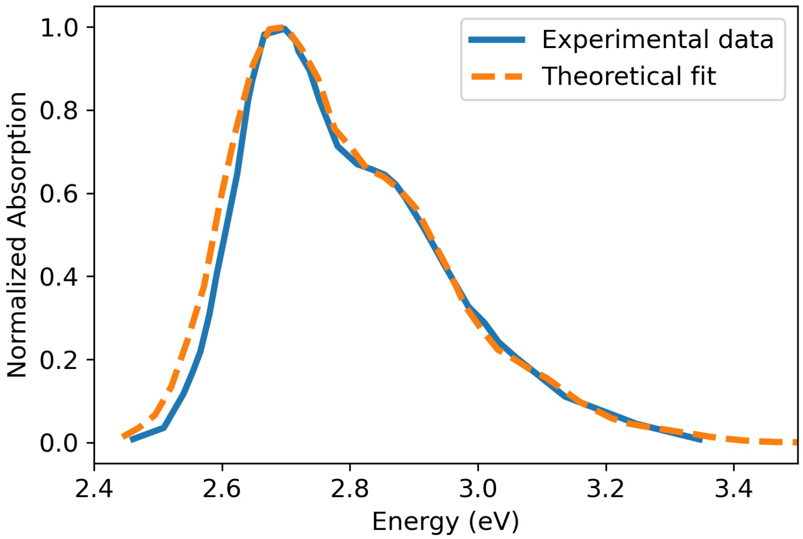

Figure S10: *Comparison between the experimental absorption spectrum and the fit with the Holstein model for molecularly dissolved s-CBT in the solvent THF.*

We focus at first on the absorption of molecularly dissolved (non-aggregated) chromophores in solution (Fig. S10). We assume each chromophore to be initially in both the vibrational and electronic ground state. This condition holds for $k_B T << \omega_0$. The chromophore interaction with the electromagnetic field coming at frequency ω follows from Fermi's Golden Rule:[12]

$$A(\omega) = \sum_{n}^{N_{\text{mol}}} \sum_{\nu}^{N_{\text{vib}}} |\Phi_n^{\mu} f_{0\nu}|^2 \cdot \delta(\Omega_{00} + \nu\omega_0 - \omega), \tag{S12}$$

where $A(\omega)$ denotes the absorption at incident frequency ω, δ is the Dirac delta function, and Ω_{00} is the electronic transition energy of the chromophores. We introduce line broadening in

two ways: First, from experimental data we know the dephasing time for excited states to lie within 10-100 fs.[20–22] We therefore convolute the spectrum with a Lorentzian lineshape with linewidth $\gamma = 25$ meV. The residual inhomogeneous broadening is due to the random distribution of Ω_{00}.

Absorption spectrum of the aggregate

We fit the inter-site electronic coupling by employing the 'exciton1d' software package by Hetstand (https://github.com/nicholashestand/exciton1d)[23] to reproduce the experimental absorption spectrum of aggregated s-CBT in n-dodecane solution (Fig. S11). These simulations are done for an aggregate containing 50 chromophores, using 5 vibrational states within the TPA. This results in a nearest neighbor coupling $J_{n,n+1} = 0.113$ eV.

We note that the code does not implement static disorder in the site energies, so that the linewidth represents a purely inter-aggregate static broadening. We find a broadening linewidth $\sigma_{Max} \sim J_{n,n+1} = 0.113$ eV to fit nicely the experimental absorption measurement. This value σ_{Max} should be considered an upper limit to the static disorder: in the absence of intra-chain static disorder, the absorption peaks from one aggregate are well defined and are broadened by the inter-chain disorder. For a realistic system the intra-chain static disorder would broaden already the peak structure of the single chain absorption spectrum, and the inter-chain disorder would broaden the absorption features further.

All the fitted parameters are summarized in Tab. S1. We mention that our code also implements the approach of Saikin *et al.*[24] which better captures the C_3-symmetry of the CBT core. The model considers an extended tripole characterized by the presence of three transition charges positioned on the vertices of an effective equilateral triangle.[24] Fitting the free parameters of this model to the spectra results in very similar nearest-neighbor coupling.

We note that the electronic transition energy of aggregates Ω^*_{00} is 0.3601 eV lower than

Table S1: Parameters extracted from fitting the absorption spectra of non-aggregated and aggregated chromophores.

Symbol	Description	Value(s)
Ω_{00}	Electronic transition energy of non-aggregated chromophore	2.6944 eV $\sim$ 21732 cm^{-1}
Ω_{00}^{*}	Electronic transition energy of aggregates	2.3344 eV $\sim$ 18828 cm^{-1}
ω_0	Vibrational energy	0.1938 eV $\sim$ 1563 cm^{-1}
λ^2	Huang-Rhys factor	0.5625
$J_{n,n+1}$	Unscaled nearest neighbor coupling	0.113 eV $\sim$ 906 cm^{-1}
σ_{Mol}	Inhomogenous broadening of non-aggregated chromophores	0.07575 eV $\sim$ 611 cm^{-1}
σ_{Max}	Upper limit to the aggregate homogeneous broadening	0.113 eV $\sim$ 906 cm^{-1}
γ	Homogeneous broadening	0.025 eV $\sim$ 202 cm^{-1}

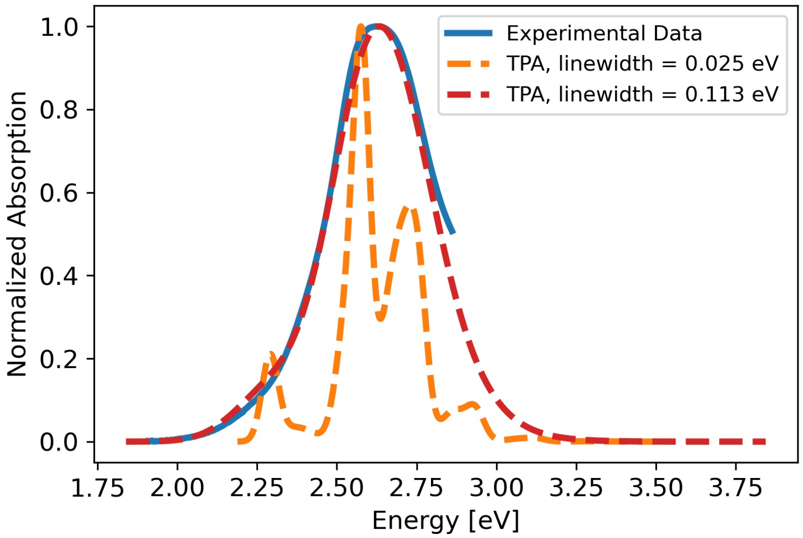

Figure S11: *Comparison between the experimental absorption spectrum of aggregated s-CBT in n-dodecane and the Holstein model in the TPA using* $J_{n,n+1} = 0.113\,eV$ *smeared with different linewidths.*

that of the non-aggregated chromophore Ω_{00}. This shift is known as the *gas-to-crystal shift*[11] and it arises due to the ability of neighbouring chromophores to stabilize the electronic ground and excited states.

Influence of high frequency vibrations on transport

In principle, accounting for vibrations should give a more accurate description of the physical system due to the multiple hopping pathways that the excitons can undergo. However, in practice we generally find that the inclusion of high-energy vibrational states (see Tab. S1) does not qualitatively change the picture discussed in the main text.

In our simulations, on aggregates with the same site-energy landscape, the inclusion of vibrations increases the diffusion exponent by around 2-5%, with negligible differences in terms of the time evolution of the propagation profile. Since the vibrational energy is quite large compared to $J_{n,n+1}$, the higher lying vibrational states do not hybridize much with the lowest vibrational state. Furthermore, due to the thermal weight from the Boltzmann distribution, the higher lying states are virtually always empty, thereby contributing less to the dynamics.

References

(1) Haedler, A. T.; Kreger, K.; Issac, A.; Wittmann, B.; Kivala, M.; Hammer, N.; Köhler, J.; Schmidt, H.-W.; Hildner, R. Long-range energy transport in single supramolecular nanofibres at room temperature. *Nature* **2015**, *523*, 196–199.

(2) Haedler, A. T.; Beyer, S. R.; Hammer, N.; Hildner, R.; Kivala, M.; Köhler, J.; Schmidt, H.-W. Synthesis and photophysical properties of multichromophoric carbonyl-bridged triarylamines. *Chem. Eur. J.* **2014**, *20*, 11708–11718.

(3) Haedler, A. T.; Meskers, S. C. J.; Zha, R. H.; Kivala, M.; Schmidt, H.-W.; Meijer, E. W.

Pathway complexity in the enantioselective self-assembly of functional carbonyl-bridged triarylamine trisamides. *J. Am. Chem. Soc.* **2016**, *138*, 10539–10545.

(4) Wittmann, B.; Wenzel, F. A.; Wiesneth, S.; Haedler, A. T.; Drechsler, M.; Kreger, K.; Köhler, J.; Meijer, E. W.; Schmidt, H.-W.; Hildner, R. Enhancing long-range energy transport in supramolecular architectures by tailoring coherence properties. *J. Am. Chem. Soc.* **2020**, *142*, 8323–8330.

(5) Wittmann, B.; Biskup, T.; Kreger, K.; Köhler, J.; Schmidt, H.-W.; Hildner, R. All-optical manipulation of singlet exciton transport in individual supramolecular nanostructures by triplet gating. *Nanoscale Horiz.* **2021**, *6*, 998–1005.

(6) Wittmann, B.; Wiesneth, S.; Motamen, S.; Simon, L.; Serein-Spirau, F.; Reiter, G.; Hildner, R. Energy transport and light propagation mechanisms in organic single crystals. *The Journal of Chemical Physics* **2020**, *153*, 144202.

(7) Xu, D.; Mandal, A.; Baxter, J. M.; Cheng, S.-W.; Lee, I.; Su, H.; Liu, S.; Reichman, D. R.; Delor, M. Ultrafast imaging of polariton propagation and interactions. *Nature Commun.* **2023**, *14*, 3881.

(8) Pandya, R. et al. Microcavity-like exciton-polaritons can be the primary photoexcitation in bare organic semiconductors. *Nature Commun.* **2021**, *12*, 6519.

(9) Thomas, P. A.; Menghrajani, K. S.; Barnes, W. L. Cavity-free ultrastrong light-matter coupling. *J. Chem. Phys. Lett.* **2021**, *12*, 6914–6918.

(10) Philpott, M. R. Calculation of the exciton band structure of the 3800- and 2500-{*aa* singlet transitions of crystalline anthracene. *The Journal of Chemical Physics* **1971**, *54*, 111–113.

(11) Spano, F. C. The spectral signatures of Frenkel polarons in h- and j-aggregates. *Acc. Chem. Res.* **2010**, *43*, 429–439.

(12) Hestand, N. J.; Spano, F. C. Expanded theory of h- and j-molecular aggregates: the effects of vibronic coupling and intermolecular charge transfer. *Chem. Rev.* **2018**, *118*, 7069–7163.

(13) Stradomska, A.; Petelenz, P. Intermediate vibronic coupling in sexithiophene single crystals. *The Journal of Chemical Physics* **2009**, *130*.

(14) Malyshev, A. V.; Díaz, E.; Domínguez-Adame, F.; Malyshev, V. A. Effects of the environment on the electric conductivity of double-stranded dna molecules. *Journal of Physics: Condensed Matter* **2009**, *21*, 335105.

(15) Weiss, U. *Quantum dissipative systems*; World Scientific, 2012.

(16) Vlaming, S. M.; Malyshev, V. A.; Knoester, J. Nonmonotonic energy harvesting efficiency in biased exciton chains. *J. Chem. Phys.* **2007**, *127*, 154719.

(17) Bednarz, M.; Malyshev, V. A.; Knoester, J. Intraband relaxation and temperature dependence of the fluorescence decay time of one-dimensional frenkel excitons: the pauli master equation approach. *J. Chem. Phys.* **2002**, *117*, 6200–6213.

(18) Sidje, R. B. Expokit. *ACM Transactions on Mathematical Software* **1998**, *24*, 130–156.

(19) Chuang, C.; Lee, C. K.; Moix, J. M.; Knoester, J.; Cao, J. Quantum diffusion on molecular tubes: universal scaling of the 1d to 2d transition. *Phys. Rev. Lett.* **2016**, *116*.

(20) Grégoire, P.; Vella, E.; Dyson, M.; Bazán, C. M.; Leonelli, R.; Stingelin, N.; Stavrinou, P. N.; Bittner, E. R.; Silva, C. Excitonic coupling dominates the homogeneous photoluminescence excitation linewidth in semicrystalline polymeric semiconductors. *Phys. Rev. B* **2017**, *95*, 180201.

(21) Bolzonello, L.; Fassioli, F.; Collini, E. Correlated fluctuations and intraband dynamics

of j-aggregates revealed by combination of 2des schemes. *J. Phys. Chem. Lett.* **2016**, *7*, 4996–5001.

(22) Kriete, B.; Bondarenko, A. S.; Alessandri, R.; Patmanidis, I.; Krasnikov, V. V.; Jansen, T. L. C.; Marrink, S. J.; Knoester, J.; Pchenichnikov, M. S. Molecular versus excitonic disorder in individual artificial light-harvesting systems. *J. Am. Chem. Soc.* **2020**, *142*, 18073–18085.

(23) Hetstand, N. Exciton1d. `https://github.com/nicholashestand/exciton1d`, 2017.

(24) Saikin, S. K.; Shakirov, M. A.; Kreisbeck, C.; Peskin, U.; Proshin, Y. N.; Aspuru-Guzik, A. On the long-range exciton transport in molecular systems: the application to h-aggregated heterotriangulene chains. *J. Phys. Chem. C* **2017**, *121*, 24994–25002.
